# Cutaneous delivery of bioactive components from a rice bran oil nanoemulsion and their biodistribution in porcine and human skin

**DOI:** 10.1016/j.ijpx.2026.100543

**Published:** 2026-04-13

**Authors:** Erga Syafitri, Aka Yoann-André Kouassi, Claudia Prezioso, Yogeshvar N. Kalia

**Affiliations:** a School of Pharmaceutical Sciences, University of Geneva, Geneva, Switzerland; b Institute of Pharmaceutical Sciences of Western Switzerland, University of Geneva, Geneva, Switzerland; c Department of Pharmacy, Faculty of Science, Institut Teknologi Sumatera, South Lampung, Lampung, Indonesia; d Department of Food and Drug, Università degli Studi di Parma, Parma, Italy

**Keywords:** Rice bran oil, Natural products, Skin delivery, Nanoemulsions, Biodistribution profile, Follicular delivery

## Abstract

Rice bran oil (RBO) is rich in antioxidants but the high lipophilicity and low concentration of key active components complicate its formulation for topical delivery. The objective of this study was to develop and to optimize an RBO-based nanoemulsion (RBO-NE) and to investigate the cutaneous delivery of three major bioactive components: 24-methylene cycloartanyl ferulate (24-MCF), γ-tocotrienol, and β-sitosterol. A validated UHPLC-MS/MS method with an internal standard was used to quantify the three molecules. The RBO-NE composition (10% RBO, 5% surfactant mix, 85% ultrapure water) was selected using a pseudo-ternary phase diagram, and ultrasonication was used to produce small globules (222 ± 2 nm, PDI 0.287), which were physically stable for three months at 4 °C. The cutaneous biodistribution of 24-MCF, γ-tocotrienol, and β-sitosterol (after consideration of endogenous β-sitosterol) was determined under infinite and finite dose conditions. Follicular targeting was evaluated by comparing delivery to pilosebaceous unit (PSU)-containing biopsies and control samples. Under infinite dose conditions, RBO-NE increased deposition of the three molecules compared to the control (RBO dispersed in hydroxypropyl-methylcellulose), with preferential accumulation in the epidermis. Delivery to the PSU from RBO-NE was approximately twice that to non-PSU containing skin samples; in contrast, the control showed no difference. Under finite dose conditions, deposition of 24-MCF and γ-tocotrienol in porcine skin was slightly superior to that in human skin. Cutaneous biodistribution studies demonstrated that (i) nanoemulsions could deliver highly lipophilic antioxidants from RBO to the epidermis and dermis, and (ii) the approach enabled simultaneous determination of the penetration profile of multiple RBO components.

## Graphical abstract

**Figure:**
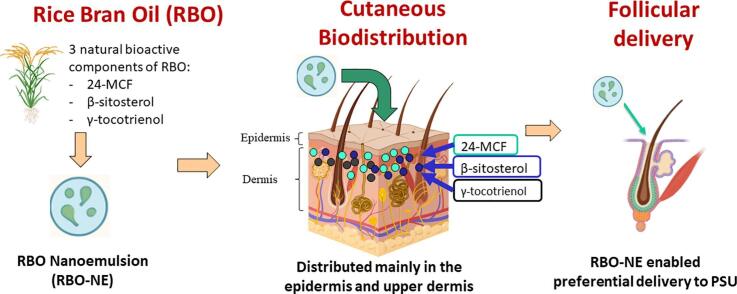

## 1 Introduction

Nanoemulsions are kinetically stable, submicron-sized dispersions composed of two immiscible liquids stabilized by surfactants and co-surfactants (if needed). Typically ranging between 100 and 500 nm in droplet size, they exhibit superior properties over conventional emulsions, such as higher surface area, better drug-loading efficiency, and have been reported to increase topical delivery (Machado et al., 2022; Souto et al., 2022). These advantages have positioned nanoemulsions as interesting candidates for the formulation and delivery of lipophilic compounds for various dermatological and cosmetic applications. Notably, the low surfactant concentration required for nanoemulsions compared to microemulsions reduces the potential for skin irritation, enhancing their safety profile for long-term topical use (Gupta et al., 2016).

The potential applications of natural oils in dermatology and their inclusion in skincare products have garnered increasing interest in recent years and rice bran oil (RBO), a by-product of rice milling, has emerged as a particularly promising candidate – especially in Asian countries (e.g. India, Indonesia, and Japan) – due to its many bioactive components (Pali, 2013). Several studies have reported the effectiveness of RBO-based formulations in promoting skin health, reducing oxidative damage, and serving as a UV protective agent (Bernardi et al., 2011; Iqbal et al., 2005; Patel and Naik, 2004; Rigo et al., 2015; Thanonkaew et al., 2015). Extracted from the outer husk of rice (*Oryza sativa*), RBO is rich in unsaponifiable fractions such as phytosterols, tocopherols, tocotrienols, and a unique blend of ferulic acid esters known as γ-oryzanol (Table 1). These components contribute to potent antioxidant, anti-inflammatory, and anticancer activities, making it a valuable candidate for skin protection and rejuvenation (Islam et al., 2008; Liu et al., 2021; Tian et al., 2024).

**Table 1:** Natural products contained in rice bran oil.

Compounds/Class	Components	Percentage (%, g/100 g)	References
γ-oryzanol	24-methylene cycloartanyl ferulate	0.4–0.8	(Lu et al., 2014)
	Cycloartenyl ferulate	0.2–0.5	
	Campesteryl ferulate	0.1–0.4	
	Sitosteryl ferulate	0.1–0.3	
	Stigmastanyl ferulate	0.1–0.3	
Tocols	γ-tocotrienol	0.07–0.08	(Liu et al., 2019; Sultana et al., 2021)
	α-tocopherol	0.04	
Phytosterols	β-sitosterol	0.9–1	(Pali, 2013; Sultana et al., 2021; Wongwaiwech et al., 2023)
	Campesterol	0.02–0.03	
	Stigmasterol	0.01–0.02	
Squalene		0.4	(Liu et al., 2019)

γ-oryzanol was initially identified as a single compound with a unique CAS Identifier (11042–64-1). However, further research revealed that γ-oryzanol is actually a mixture of several molecules with similar properties (Table 1) (Fang et al., 2003). It is composed of a series of ferulic acid esters of plant sterols and triterpenoids, which differentiates it from other vegetable oils. It is of particular interest due to its capacity to scavenge reactive oxygen species (ROS), inhibit lipid peroxidation, and modulate inflammatory pathways. Several methods, including HPLC-UV and UHPLC-MS/MS, have been used to separate these compounds (Fang et al., 2003; Liu et al., 2013; Lu et al., 2014).

**Fig. 1:**
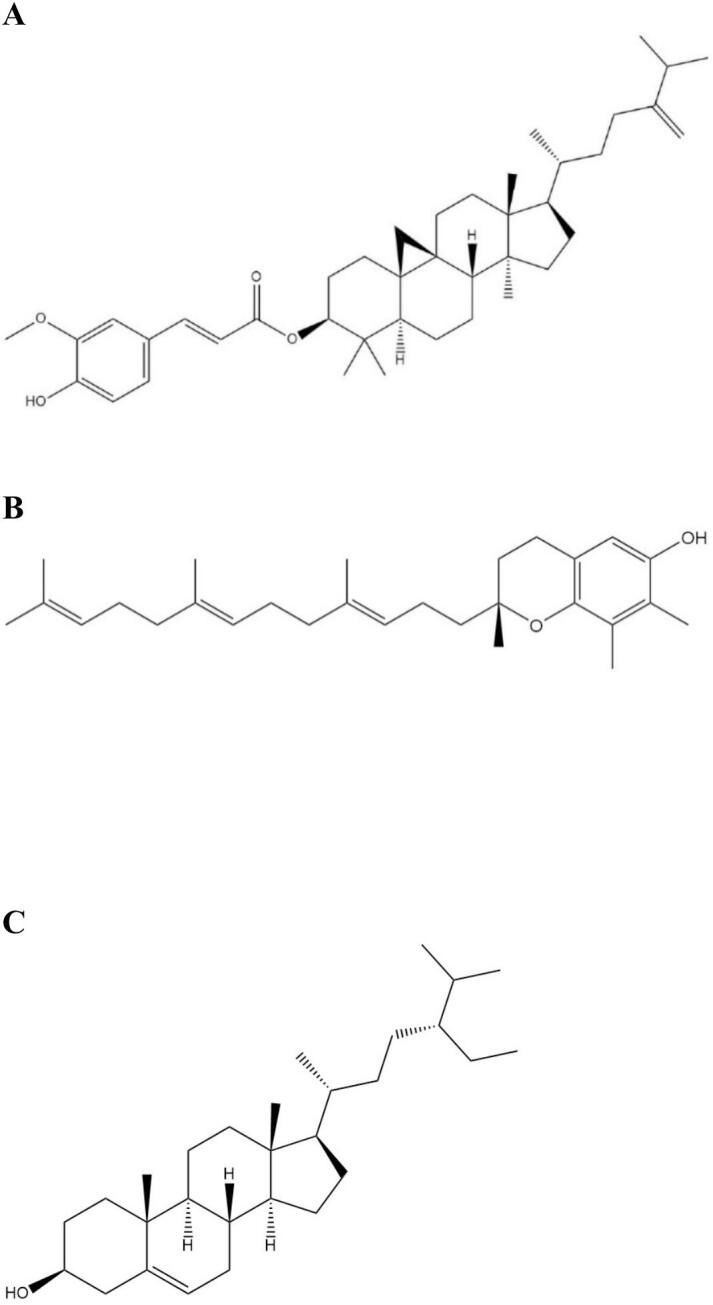
Structure of (A) 24-methylene cycloartanyl ferulate (24-MCF), (B) γ-tocotrienol and (C) β-sitosterol. Chemical structures were drawn using ChemDraw Professional (64-bit; 22.2.0.3300).

In this study, we focused on the delivery of three key RBO components– (i) 24-methylene cycloartanyl ferulate (24-MCF) as a representative of the γ-oryzanol “group”, (ii) γ-tocotrienol as a representative of the tocol family, and (iii) β-sitosterol as a representative of the sterol group (Fig. 1). 24-methylenecycloartanyl ferulate (24-MCF) is a key component of γ-oryzanol and has been identified as having the most potent antioxidant activity among the oryzanol esters (Islam et al., 2009; Juliano et al., 2005) (Fig. 1A). Tocotrienols, which are vitamin E isoforms predominantly found in RBO, palm, and cereal grains, have demonstrated superior antioxidant and anti-inflammatory efficacy compared to tocopherols, particularly in the context of skin aging and photo-protection (Ghazali et al., 2022). Notably, γ-tocotrienol (Fig. 1B) has been shown to be as potent as α-tocotrienol in neutralizing free radicals in vitro, while α-tocotrienol has demonstrated antioxidant capacity 40–60 times greater than that of α-tocopherol in microsomal systems (Palozza et al., 2006; Serbinova et al., 1991). Phytosterols such as β-sitosterol (Fig. 1C) also contribute to RBO's activity. Structurally similar to cholesterol but derived exclusively from plant sources, these compounds exhibit anti-inflammatory, immunomodulatory, and anticancer effects (Shen et al., 2024). RBO contains a higher proportion of β-sitosterol compared to other vegetable oils (Sawadikiat and Hongsprabhas, 2014).

The physicochemical properties of these three natural products (NP) are presented in Table 2.

**Table 2:** Properties of the three main natural products contained in RBO.

	24-MCF	γ-tocotrienol	β-sitosterol
Molecular Formula	C_41_H_60_O_4_	C_28_H_42_O_2_	C_29_H_50_O
Molecular weight (g/mol)	616.9	410.6	414.7
Log K_o/w_⁎	12.6	8.07	8.14
Content in RBO ⁎⁎	0.2 g/100 g oil (0.3 mmol/100 g oil)	0.04 g/100 g oil (0.2 mmol/100 g oil)	0.09 g/100 g oil (0.12 mmol/100 g oil)
Pharmacological activities	Antioxidant, anti-inflammatory agent (Islam et al., 2009)	Strong antioxidant, photo-protection (Ghazali et al., 2022)	Antiinflammatory, immunomodulator (Valerio and Awad, 2011)

⁎ Predicted using ChemDraw Professional (64-bit; 22.2.0.3300).

⁎⁎ Measured experimentally, see Section 2.3 and Supplementary Data, Section 1.

To date, research into the topical application of RBO has mainly concerned formulation development and evaluations of the effect on skin, with little focus on the cutaneous penetration of individual bioactive RBO components. Knowledge of the amounts present (and hence the concentrations) as a function of penetration depth and the anatomical region is critical in understanding whether a given component contributes to the presence of a physiologic effect. This is obviously important for formulation optimization and to establish safety. To address this, we have combined cryotome sectioning and precise quantification using UHPLC-MS/MS, enabling accurate mapping of the cutaneous biodistribution in the stratum corneum, viable epidermis, and dermis (Darade et al., 2023; Gou et al., 2022; Quartier et al., 2021; Syafitri and Kalia, 2025). The method provides a high-resolution, quantitative framework to assess the localization of topically applied compounds within discrete skin compartments. It has been validated and applied in various cutaneous delivery studies but has not yet been exploited to explore biodistribution profiles following the application of complex natural products. To further investigate penetration pathways following nanoemulsion application, follicular delivery was assessed using a punch biopsy-based approach. This method enables quantitative comparison of pilosebaceous unit (PSU)-containing biopsies and adjacent PSU-free controls, providing direct evidence of follicular accumulation (Kandekar et al., 2018; Lapteva et al., 2015). These two techniques provide a comprehensive overview of molecular distribution in skin and appendageal structures.

In this context, the present study aimed to systematically investigate the simultaneous delivery and cutaneous biodistribution of three key natural products (NP) from RBO – 24-MCF, γ-tocotrienol, and β-sitosterol – after formulation in a nanoemulsion for topical application. The compounds were selected based on their physicochemical properties, bioactivity, and relevance to RBO authentication. The specific objectives were (a) to develop and to validate a robust bioanalytical method for the simultaneous quantification of these molecules using an internal standard (cholesterol-D6), (b) to develop and to optimize an RBO nanoemulsion (RBO-NE) formulation and to determine its stability over 3 months by assessing droplet size and PDI, (c) to determine the cutaneous delivery and biodistribution of 24-MCF, γ-tocotrienol and β-sitosterol in porcine and human skin using infinite and finite dose conditions, and (d) to investigate the follicular delivery of each NP released from RBO-NE and provide insight into the potential for targeted delivery to appendageal structures.

## 2 Materials and methods

### 2.1 Materials

Rice bran oil was purchased from Gustav Heess GmbH (Leonberg, Germany). 24-MCF was bought from ChemFaces (Wuhan, China). β-sitosterol was purchased from MedChem Express (Lucerne, Switzerland). Cholesterol-D6 and γ-tocotrienol were purchased from Cayman Chemical (Michigan, USA). Sorbitan oleate, Tween 80, Cremophor EL (HLB 12–14), hydroxypropyl methylcellulose (HPMC, ∼26 kDa), formic acid (MS grade), isopentane, sodium chloride and Dulbecco's phosphate-buffered saline (without calcium chloride and magnesium chloride; DPBS) were purchased from Sigma Aldrich (Buchs, Switzerland). Bovine serum albumin (BSA) was purchased from Axon Lab (Baden-Dättwil, Switzerland). Acetonitrile LC/MS grade was obtained from Fisher Scientific (Reinach, Switzerland) and acetone was obtained from Acros Organics (Geel, Belgium). O.C.T. mounting medium was purchased from VWR Chemicals (Leuven, Belgium). Labrasol® ALF and Transcutol® P were kindly provided by Gattefossé SAS (Saint–Priest, France). Nylon filters were purchased from VWR (Nyon, Switzerland). Ultrapure water (Millipore Milli-Q Gard 1 Purification Pack resistivity >18 MΩ.cm; Zug, Switzerland) was used to prepare all solutions. All other chemicals were of at least analytical grade.

### 2.2 Analytical methods

UHPLC with tandem mass spectrometry detection (UHPLC-MS/MS) was used to quantify 24-MCF, γ-tocotrienol, and β-sitosterol in RBO, the different formulations, and their cutaneous/follicular deposition, using cholesterol-D6 as an internal standard (IS). The samples were analyzed using a Waters Acquity UPLC® system and Waters XEVO TQ-S micro triple quadrupole mass spectrometer (Baden-Dättwill, CH). The chromatographic column used was an XBridge® BEH-C8 column (2.1 × 50 mm, 2.5 μm) in tandem with an XBridge BEH C8 XP VanGuard Cartridge (2.1 mm × 5 mm, 2.5 μm) maintained at 40 °C. The gradient separation was performed using a mobile phase of (A) Acetonitrile +0.1% Formic Acid and (B) Milli-Q Water +0.1% Formic Acid. The flow rate was 0.4 mL/min with an injection volume of 10 μL. The gradient method is presented in Table 3. Each injected sample contained IS at a concentration of 100 ng/mL.

**Table 3:** UHPLC-MS/MS gradient programme.

Time (min)	Flow rate (mL/min)	A%	B%
0	0.4	95	5
1	0.4	25	75
4	0.4	0	100
4.5	0.4	0	100
4.6	0.4	95	5
6	0.4	95	5

Mass spectrometric detection was performed with electrospray ionization using Multiple Reaction Monitoring (MRM) mode, and the data obtained were analyzed using Masslynx V4.2 SCN1040 software. The UHPLC-MS/MS method was validated in solvent, and porcine and human skin matrices using ICH Bioanalytical Method Validation guidelines (complete details are provided in the Supplementary Data, Section 2). The different MS/MS settings are presented in Table 4.

**Table 4:** MS/MS settings for detection of 24-MCF, γ-tocotrienol, β-sitosterol, and cholesterol-D6 (Internal Standard; IS).

Parameters	24-MCF	γ-Tocotrienol	β-Sitosterol	Cholesterol-D6 (IS)
Nature of parent ion	${\left[M-194+H\right]}^{+}$	${\left[M+H\right]}^{+}$	${\left[M-H_{2}O+H\right]}^{+}$	${\left[M-H_{2}O+H\right]}^{+}$
Precursor ion (*m*/*z*)	423.36	411.25	397.28	375.41
Fragmented ion (m/z)	95.01	150.98	147.8	147.08
Collision energy (V)	34	46	22	22
Cone voltage (V)	2	28	10	2
Capillary Voltage (kV)	3	3	3	3
Source temperature (°C)	150	150	150	150
Desolvation temperature (°C)	450	450	450	450
Desolvation gas flow (L/h)	900	900	900	900
Cone gas flow (L/h)	0	0	0	0
LM resolution 1	15	15	15	15
HM resolution 1	15	15	15	15
Ion energy 1 (V)	0.5	0.5	0.5	0.5
LM resolution 2	15	15	15	15
HM resolution 2	15	15	15	15
Ion energy 2 (V)	0.5	0.5	0.5	0.5
Retention Time (min)	4.19	2.95	3.65	3.27

### 2.3 Quantification of 24-MCF, γ-tocotrienol and β-sitosterol in RBO

20 mg of RBO was accurately weighed and transferred to a 5 mL volumetric flask, then dissolved in n-hexane to reach a final volume of 5 mL. This solution was diluted 100-fold with acetonitrile (ACN). The sample was then filtered through a 0.22 μm nylon filter. The filtrate was then mixed 1:1 (*v*/v) with an IS solution (200 ng/mL in ACN) to obtain a final IS concentration of 100 ng/mL. The samples were analyzed using UHPLC-MS/MS for quantification of 24-MCF, γ-tocotrienol and β-sitosterol in RBO. The method was validated, and a detailed protocol is provided in the Supplementary Data, Section 3. The NP content was expressed as % amount (g/100 g RBO).

### 2.4 Development of an RBO-based nanoemulsion formulation

Different surfactants and co-surfactants were selected and tested for this study, including Labrasol® (HLB 12), Tween 80 (HLB 15), Cremophor EL (HLB 13), Transcutol® and sorbitan oleate (HLB 4.3), totalling 10% (*w*/w) as emulsifiers. Their compatibility with RBO was evaluated, and the chosen surfactants, combined at different ratios (4:1, 1:1, and 1:4), were used to construct pseudo-ternary phase diagrams using the water titration method, as described in our previous publication (Syafitri and Kalia, 2025). Turbidity observed during water addition indicated biphasic regions, while the clear, translucent mixture signified a monophasic region. The diagram was generated using TernaryPlot software. A suitable composition from the biphasic region, identified as a coarse emulsion (EM), was chosen for further optimization.

The size of the EM globules was reduced to create an RBO-based nanoemulsion (RBO-NE) by applying a probe ultrasonicator (Branson Sonifier® SFX 250) for several minutes in an ice bath. Optimization involved varying component ratios, and sonication times (30 s, 45 s, and 60 s) to achieve the target droplet size and stability of the RBO-NE.

### 2.5 Characterization of the RBO-NE formulation

#### 2.5.1 Size and zeta potential determination

The hydrodynamic diameter (Z_av_), polydispersity index (PDI), and number-weighted diameter (d_n_) were assessed using dynamic light scattering (DLS) with a Zetasizer Nano S (Malvern Instruments Ltd.; Malvern, UK). Zeta potential was measured via electrophoretic light scattering with a Zetasizer Nano ZS (Malvern Instruments Ltd.; Malvern, UK). Measurements were conducted at a 90° angle and at a temperature of 25 °C. The conductivity for all tests ranged from 0.01 to 1 mS/cm^2^. All data were derived from 15 runs of 3 measurements each.

#### 2.5.2 Measurement of pH

The pH of RBO-NE was determined using a Mettler Toledo® pH meter. The measurement was done in triplicate at a temperature of 25 °C.

#### 2.5.3 Morphology

The morphology of RBO-NE was analyzed by transmission electron microscopy (Talos L120C 1549b, Thermo Fisher Scientific, Switzerland) with negative staining. RBO-NE was diluted 10 times before measurement (particle size measurements using the Zetasizer showed no difference in size before and after dilution). 5 μL of the diluted formulation was placed onto an ionized, carbon-coated copper grid (0.3 Torr, 400 V for 20 s). The grid was then briefly exposed for 1 s with a 100 μL drop of saturated uranyl acetate aqueous solution, followed by another 30 s with an additional 100 μL drop. Excess staining solution was removed, and the grid was dried at room temperature before measurement.

#### 2.5.4 Determination of 24-MCF, γ-tocotrienol and β-sitosterol concentration

RBO-NE was diluted 1000-fold with ACN and then filtered through a 0.22 μm nylon filter. The diluted sample was then mixed 1:1 (*v*/v) with an IS solution (200 ng/mL in ACN) to obtain a final IS concentration of 100 ng/mL. The prepared sample was analyzed using UHPLC-MS/MS, and the concentration of each NP was expressed using the following equation.

$$\mathrm{NP}\;\text{content}\;\left(\mathrm{mM}\right)=\frac{\text{mass of}\;\mathrm{NP}\;\text{in the formulation}\;\left(\text{mmol}\right)}{\text{volume of the formulation}\;\left(L\right)}$$

The concentration of each NP in RBO-NE was determined monthly for a period of 3 months.

#### 2.5.5 Physical stability study

The physical stability of RBO-NE was evaluated with respect to centrifugation, freeze-thaw cycles, and storage stability. The centrifugation test was conducted at RCFs of 581 ×g and 2325 ×g for 15 min, while storage stability was assessed by keeping the formulation at 4 °C for three months (Felipe et al., 2024). In the freeze-thaw test, the RBO-NE formulation was kept in glass vials at 4 °C for 24 h, then at room temperature for 24 h to complete one cycle. This cycle was repeated three times over six days. The formulations were examined to measure the globule size and PDI before and after the stability test.

### 2.6 Evaluation of cutaneous delivery in vitro

#### 2.6.1 Skin preparation

Porcine ear skin was obtained from a local abattoir (CARRE; Rolle, CH). All skin samples were washed under cold running water and processed with a Zimmer air dermatome (Münsingen, CH) set to a thickness of 1000 μm. The thickness of each sample was then measured with a micrometre (Mitutoyo 2046S, Japan) and precisely recorded. The average thickness across all samples was 993 ± 77 μm. Hair was carefully shaved from the skin surface using clippers. Circular discs with a 22 mm diameter were punched out (Berg & Schmid HK 500; Urdorf, Switzerland) and stored at −20 °C for a maximum period of up to 3 months. Before testing, samples were thawed at room temperature and rehydrated by soaking in a 0.9% NaCl solution for 15 min.

Human skin samples were collected immediately after surgery from the Department of Plastic, Aesthetic, and Reconstructive Surgery at Geneva University Hospital in Switzerland. The study received approval from the Cantonal Committee for Ethics in Research (Project-ID: 2021–01578). Hypodermis and fatty tissue were carefully removed, and discs matching the permeation area were punched out using a Berg & Schmid HK 500 device (Urdorf, Switzerland). The average thickness of the human skin samples was 999 ± 64 μm. The samples were then stored in a biobank at −20 °C for a maximum period of 3 months (Darade et al., 2023).

#### 2.6.2 Quantification of endogenous β-sitosterol in porcine and human skin

Quantification of endogenous β-sitosterol was performed using samples from various donors (3 humans and 5 porcine ears). Each donor provided 3 skin replicates, totaling 9 human and 15 porcine skin samples. The skin was cut into 0.5 cm^2^ pieces and weighed accurately. Then the skin was cut into small pieces and extracted with 2.5 mL of ACN containing IS at a final concentration of 100 ng/mL. The extracts were centrifuged at 12000 rpm ((16,128 × g)) for 15 min, then filtered and analyzed using UHPLC-MS/MS. The results are provided in the Supplementary Data, Section 4.

#### 2.6.3 Cutaneous delivery and biodistribution profile

Skin samples (*n* = 6) were mounted in Franz diffusion cells (Milian SA; Meyrin, CH) with a formulation contact area of 0.8 cm^2^. The receptor compartment contained 3 mL of PBS at pH 7.4 and contained Tween 80 (0.1%, *w*/w) and was kept at 32–34 °C throughout the experiments. For infinite dose conditions, 200 μL of RBO-NE formulation (with 100 mg RBO/mL of formulation) was applied to the skin sample surface (corresponding to 25 mg of RBO/cm^2^ of skin surface). The application time for the infinite dose experiments was 8 h, using porcine skin samples. For the finite dose experiment, 8 μL of RBO-NE formulation (100 mg RBO/mL of formulation) was applied (corresponding to 1 mg of RBO/cm^2^ of skin surface). The finite dose experiments were conducted for 8, 16, and 24 h on porcine skin, while on human skin, they were performed for 8 and 24 h. A non-emulsified formulation without surfactant, comprising 100 mg/mL RBO mixed with an aqueous solution of 1% hydroxypropyl methylcellulose (HPMC), was used as a control and applied under the same conditions. This would minimize the risk of interference of the suspending agent on drug delivery. These conditions complied with the OECD guidelines (OECD, 2004; OECD, 2011). After the experiment, 1 mL of the receiver phase was withdrawn to quantify the transdermal permeation of 24-MCF, γ-tocotrienol, and β-sitosterol. Samples were mixed with 1 mL of IS solution (200 ng/mL in ACN) to obtain a final IS concentration of 100 ng/mL and to precipitate salts. After centrifugation at 12,000 rpm (16,128 ×g) for 15 min, the permeation samples were analyzed by UHPLC-MS/MS.

Upon completion of the experiments, each skin sample was carefully cleaned by washing under running PBS + Tween 80 (0.1% *w*/w) 3 times and rubbing gently with cotton buds, finally dried with soft tissue paper (validated data in Supplementary Data, Section 5). An 8 mm punch was used to separate the skin sample into two parts – an inner disc with a surface area of 0.5 cm^2^ and a remaining outer ring with an area of 0.3 cm^2^. This outer ring was subsequently cut into small pieces, and the total deposition of 24-MCF, γ-tocotrienol, and β-sitosterol was extracted by soaking the pieces in 2.5 mL of ACN containing IS at a final concentration of 100 ng/mL for 18 h with continuous stirring at room temperature. The extraction procedure was validated, and the detailed procedure is provided in the Supplementary Data, Section 6.1. The extraction samples were centrifuged at 12,000 rpm (16,128 ×g) for 15 min and filtered through a 0.22 μm nylon filter before UHPLC-MS/MS analysis.

The 0.5 cm^2^ discs were used to determine the biodistribution of 24-MCF, γ-tocotrienol and β-sitosterol concentration at different depths within the skin. These skin discs were snap-frozen in isopentane cooled by liquid nitrogen, covered with a plastic clip to prevent any 24-MCF, γ-tocotrienol and β-sitosterol from dissolving into the isopentane. The frozen skin discs were embedded in O.C.T. and fixed on a square piece of cork, with a plastic o-ring placed around the discs to prevent tissue compression and ensure a flat, frozen surface. The skin discs were then cryosectioned using a Thermo Scientific™ CryoStar™ NX70 (Reinach, Switzerland), producing ten layers, each with a thickness of 40 μm, starting from the stratum corneum down to a depth of 400 μm. Each layer and the remaining dermis were individually extracted in 250 μL of extraction solution (ACN containing IS at a final concentration of 100 ng/mL) for 24 h, followed by sonication (Branson Ultrasonic Cleanser 55100E-MT) for 20 min in an Eppendorf tube, and the amounts of 24-MCF, γ-tocotrienol, and β-sitosterol were quantified using UHPLC-MS/MS. The extraction procedure for the biodistribution samples was validated, and the detailed procedure is provided in the Supplementary Data, Section 6.2.

#### 2.6.4 Evaluation of follicular delivery in porcine skin

An infinite dose of RBO-NE formulation and control formulation (1 mg of RBO/cm^2^ of the skin surface) was applied for 8 h to the donor compartment containing porcine skin. After completing the delivery experiments, the skin was cleaned according to the protocol described above. The method, which allows direct quantification of delivery to the intact pilosebaceous unit (PSU), was previously used to investigate the ability of micelles to target follicular structures (Kandekar et al., 2018; Lapteva et al., 2015). The entire PSU was harvested with a 1 mm diameter punch (Berg & Schmid HK 500; Urdorf, Switzerland). The amounts of 24-MCF, γ-tocotrienol and β-sitosterol present in the PSU biopsies were compared with control biopsies where no PSU was present; they were extracted over 24 h using 100 μL of extraction solution (ACN containing IS at a final concentration of 100 ng/mL) at room temperature on a shaker at 500 rpm in an Eppendorf tube followed by sonication for 20 min. The samples were centrifuged at 12,000 rpm (16,128 x g) for 15 min. Supernatants were collected, and 24-MCF, γ-tocotrienol, and β-sitosterol concentrations were quantified using UHPLC-MS/MS.

### 2.7 Data analysis

Data were expressed as mean ± standard deviation (mean ± SD). Statistical differences were determined by the Mann-Whitney test for two comparisons, and the Kruskal-Wallis H test for multiple comparisons, followed by Tukey's test. The level of significance was fixed at α = 0.05.

## 3 Results and discussion

### 3.1 Development and characterization of the RBO-NE formulation

**Fig. 2:**
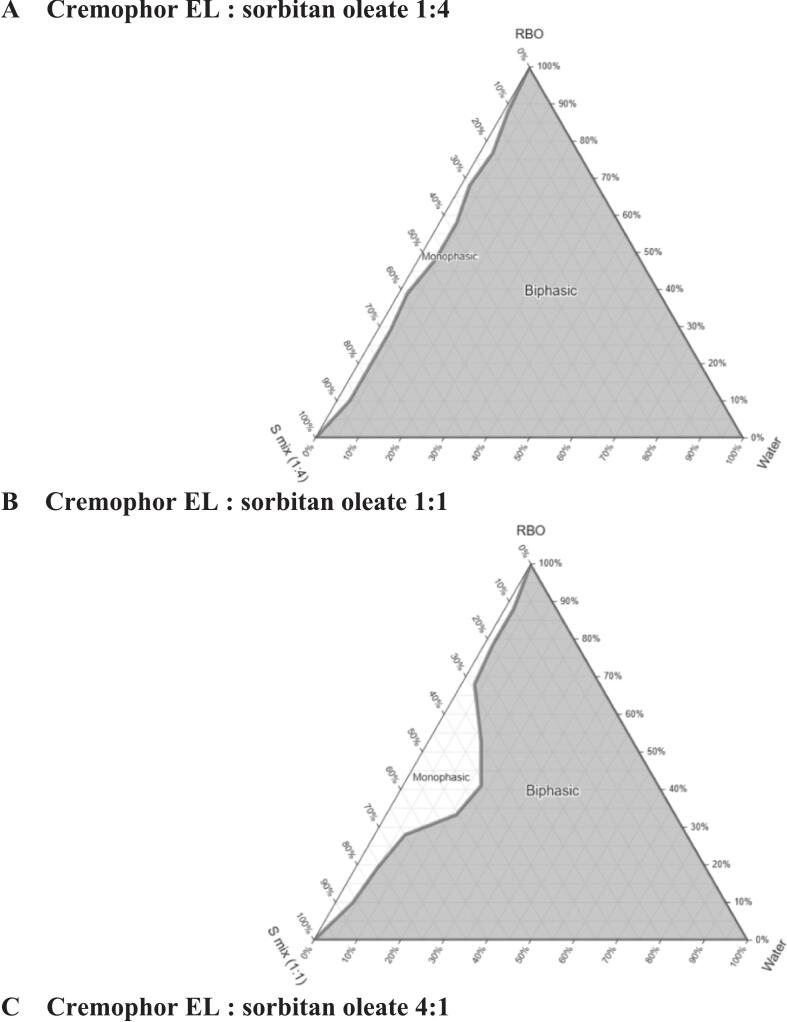

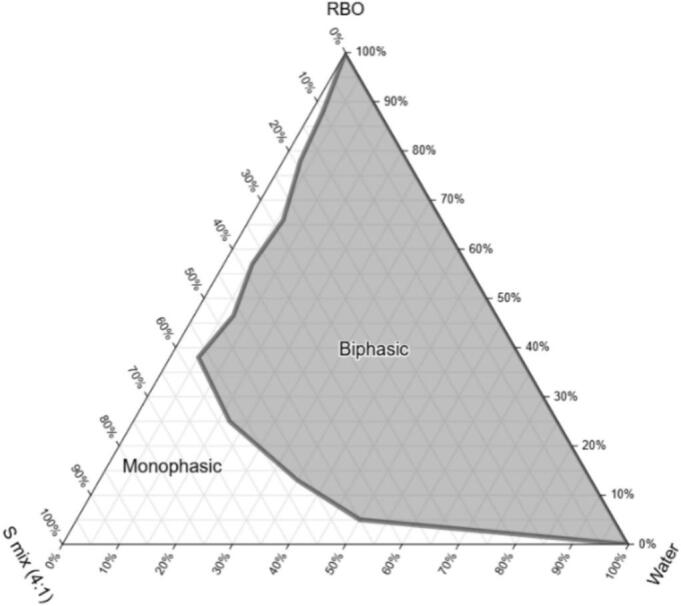
Pseudo-ternary phase diagram for RBO, Surfactant mix (Cremophor EL: sorbitan oleate) and water systems. The grey area denotes a biphasic region, and the white area is a monophasic region.

Several surfactants (Tween 80, Cremophor EL, and Labrasol®) and co-surfactants (Transcutol® and sorbitan oleate) were tested to formulate an emulsion with RBO, using a surfactant: co-surfactant ratio of 4:1. The physical appearance of the emulsion was observed for 3 days and the combination of Cremophor EL and sorbitan oleate was selected due to the homogeneity and the absence of separation (Supplementary Data, Section 7). To develop stable nanoemulsions, pseudo-ternary phase diagrams were constructed using the water titration method, with RBO as the oil phase, Cremophor EL as surfactant, sorbitan oleate as co-surfactant – S_mix_ (Cremophor EL: sorbitan oleate) ratios of 1:4, 1:1, and 4:1 – and water (Fig. 2).

The combination of S_mix_ at a 4:1 ratio (HLB mixture = 10.46) was selected for further optimization to produce the RBO-NE. Three different behaviours – depending on the water content – were observed during formulation development: (i) a water content of 10–20% produced water in oil (w/o) emulsions, (ii) increasing water content to 40–60% resulted in the formation of creamy emulsions (bicontinuous phase), and (iii) at 80% water, oil in water emulsions with a free-flowing formulation were formed (Zeng et al., 2017). Nanoemulsions were prepared using a two-step process: first, formation of a coarse emulsion (EM), then application of ultrasound to decrease droplet size.

The nanoemulsions were classified as oil-in-water (o/w) due to their high-water content, with formulations chosen from the biphasic region. EM formulations containing 80% and 85% water were selected with S_mix_ ratios 4:1. Sonication times of 30, 45, and 60 s were tested (Table 5).

**Table 5:** Optimization of RBO-NE.

Formulation	Composition				Sonication time (s)	Size characterization		
	RBO (%)	Surfactant (%)		Water (%)		Z_av_ (nm)	PDI	d_n_ (nm)
		Cremophor EL	Sorbitan Oleate					
A1	10	8	2	80	30	305	0.272	220
A2	10	8	2	80	45	302	0.286	229
A3	10	8	2	80	60	302	0.401	160
B1	10	4	1	85	30	339	0.340	192
B2	10	4	1	85	45	293	0.362	172
B3	10	4	1	85	60	222	0.287	87

The smallest droplets of 222 nm were produced with formulation B3, which contained 85% water, 10% RBO, and 5% S_mix_. The high water content and low surfactant level (5% S_mix_) in this formulation were considered likely to enhance skin compatibility and reduce irritation risk in vivo, while maintaining nanoemulsion stability. The optimal sonication time was 60 s; beyond this point, extending sonication offered minimal benefits, since droplet size reached a stability limit set by the surfactants' capacity to stabilize the interface. Thus, formulation B3 (henceforth, RBO-NE B3) was selected for subsequent experiments.

Quantification of 24-MCF, γ-tocotrienol and β-sitosterol in RBO-NE B3 revealed that 24-MCF had the highest concentration (0.27 ± 0.01 mM), followed by β-sitosterol (0.21 ± 0.03 mM), and finally γ-tocotrienol (0.09 ± 0.02 mM); the contents were in agreement with the composition of RBO. 24-MCF is present at the highest level among the three components, at 0.3 mmol/100 g oil (0.2 g/100 g of oil), followed by β-sitosterol at 0.2 mmol/100 g (0.09 g/100 g), and γ-tocotrienol at 0.12 mmol/100 g (0.04 g/100 g). The bioactive content percentage is affected by the refining process of the oil (Liu et al., 2019; Pali, 2013; Wongwaiwech et al., 2023).

**Fig. 3:**
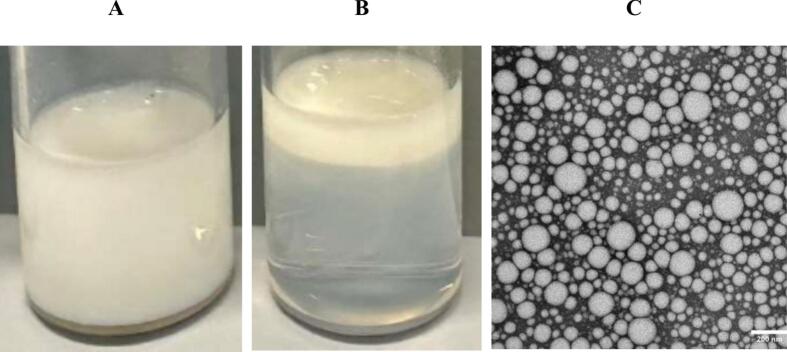
Physical observation of (A) RBO-NE B3 and (B) EM obtained after 3 days. (C) Visualization of RBO-NE B3 using TEM: scale bar 200 nm.

The physical appearance of RBO-NE B3 and EM (the coarse emulsion with the same composition as RBO-NE B3 but produced without sonication, resulting in larger droplets) is shown in Fig. 3. After 3 days, the EM (Z_av_ = 1677 nm, PDI > 1.00) became unstable and separated into two phases, whereas RBO-NE B3 (Z_av_ = 222 nm; PDI = 0.287) remained stable, retaining a monophasic, milky appearance (Fig. 3A and B). These visual observations underlined the superior stability of the nanoemulsion compared to the conventional emulsion, demonstrating its robustness against coalescence and flocculation. The zeta potential of RBO-NE B3 was measured at −17.6 ± 0.95 mV, only slightly negative, indicating the presence of little or no electrostatic stabilization.

The small droplet size of RBO-NE B3 results from the energy of the ultrasonication process, which causes cavitation – the rapid formation of bubbles in a liquid at ambient temperature under reduced pressure (Azmi et al., 2022). As droplet size decreases, attractive forces weaken more rapidly than repulsive forces, resulting in kinetic stability. The TEM image of RBO-NE B3 shows spherical droplets with uniform morphology, indicative of a well-formed and stable nanoemulsion system (Fig. 3C).

**Fig. 4:**
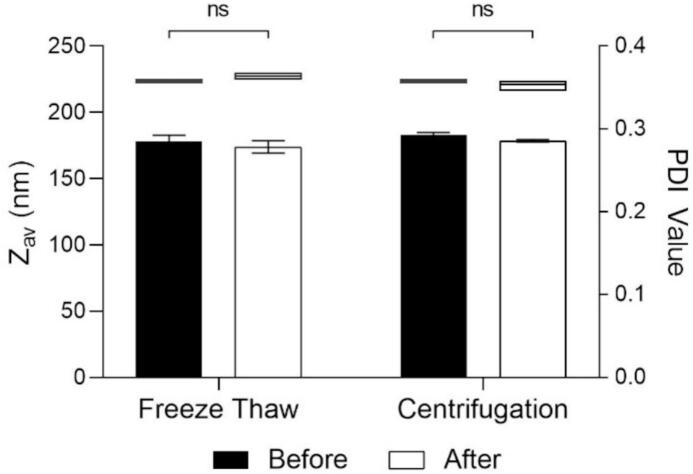
Z_av_ (floating bar) and PDI (column) of RBO-NE B3 before and after freeze-thaw and centrifugation tests (Mean ± SD, *n* = 6, *p* > 0.05, Mann-Whitney test).

No significant changes were observed in droplet size of RBO-NE B3 after freeze-thaw cycles and centrifugation and the formulation maintained a small droplet size with a narrow PDI, indicating uniformity and stability (Fig. 4). The droplet size of RBO-NE B3 before and after the freeze-thaw test was 222.3 ± 1.5 nm and 227.1 ± 2.3 nm, respectively. For the centrifugation test, Z_av_ before and after freeze-thaw test was 222.3 ± 1.5 nm and 221.1 ± 3.9 nm, respectively. The results confirmed that the optimized nanoemulsion was sufficiently stable and suitable for the subsequent cutaneous delivery studies.

**Fig. 5:**
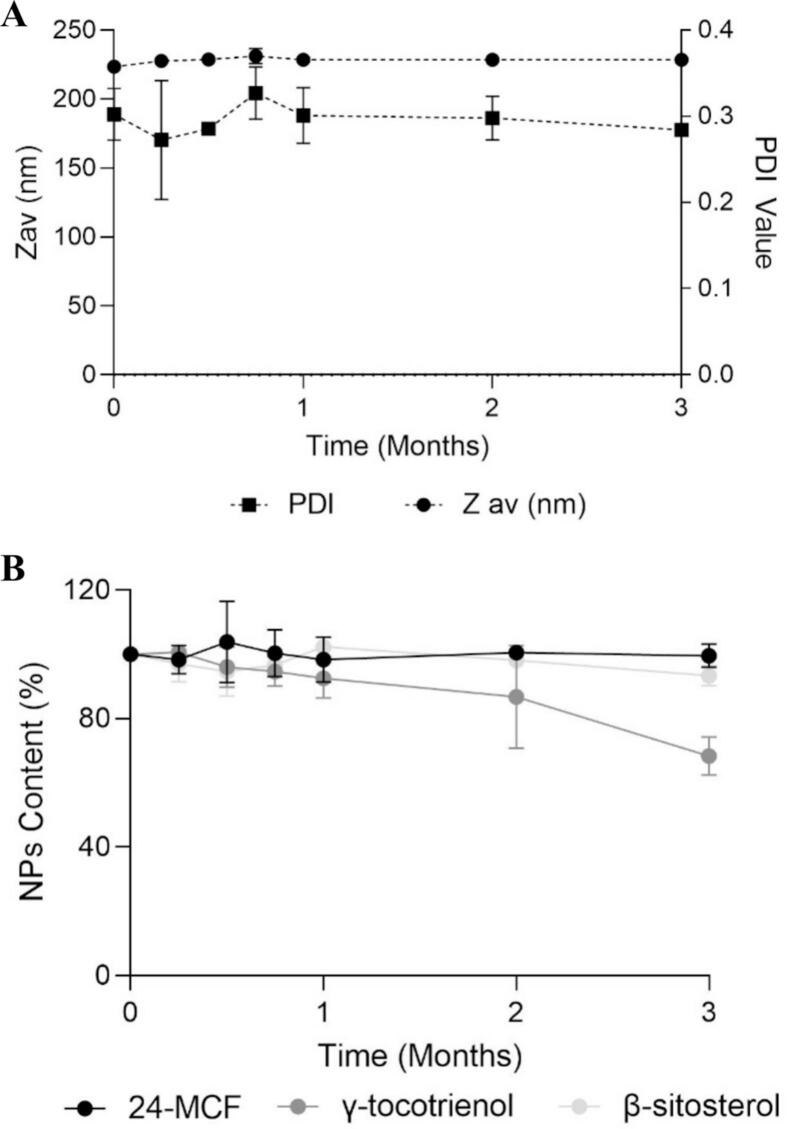
Stability of RBO-NE B3 during storage for 3 months at 4 °C, including (A) Z_av_ and PDI, (B) 24-MCF, γ-tocotrienol and β-sitosterol content (Mean ± SD, *n* = 6, p > 0.05, Kruskal Wallis test).

After storage of RBO-NE B3 for 3 months at 4 °C: the droplet size remained stable (Fig. 5A) with Z_av_ and PDI of 228 nm and 0.284, respectively (cf. initial values of 222 nm and 0.287). RBO-NE B3 maintained pH levels between 4.5 and 5 over three months (Supplementary Data, Section 8). These values are close to the skin surface pH (4–6) (Lukić et al., 2021), suggesting that RBO-NE B3 was compatible for skin application. The 24-MCF, γ-tocotrienol and β-sitosterol NP content in RBO-NE B3 was also monitored over three months (storage at 4 °C) (Fig. 5B), and the contents of 24-MCF, γ-tocotrienol, and β-sitosterol were 99.5 ± 3.6%, 68.3 ± 5.9%, and 93.3 ± 3.1% of their initial levels, respectively. While 24-MCF and β-sitosterol remained within acceptable ranges, γ-tocotrienol was outside the stability limit (85% of the initial value). This component is more susceptible to oxidation due to its structure and chemical reactivity. Notably, γ-tocotrienol can penetrate the water phase more effectively than the other two components over time, leading to interactions with oxygen and resulting in degradation (Ghazali et al., 2022; Palozza et al., 2006; Yoshida et al., 2003).

### 3.2 Evaluation of the cutaneous delivery of 24-MCF, γ-tocotrienol and β-sitosterol from RBO-NE B3 in porcine skin under infinite dose conditions

**Fig. 6:**
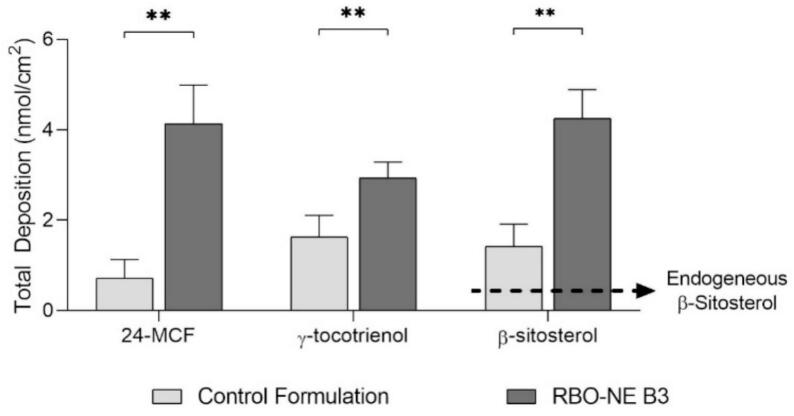
Total deposition in porcine skin of 24-MCF, γ-tocotrienol and β-sitosterol after application of RBO-NE and control formulation (10% RBO dispersed in a 1% HPMC solution) under infinite dose conditions for 8 h. The dashed line indicates endogenous β-sitosterol. (Mean ± SD, *n* = 6, ** *p* < 0.01, Mann-Whitney test).

The first cutaneous delivery experiments investigated the delivery of 24-MCF, γ-tocotrienol, and β-sitosterol after application of RBO-NE B3 and the control formulation (10% RBO dispersed in a 1% HPMC solution) under infinite dose conditions (250 μL/cm^2^). This experiment was conducted to give a general overview of the skin deposition of the different NP from RBO-NE B3 and the control and corresponds to the conditions most widely used for in vitro studies. Transdermal permeation of 24-MCF, γ-tocotrienol, and β-sitosterol was considered negligible since the concentrations present in the receiver compartment after formulation application for 8 h were below the LOD of the UHPLC-MS/MS method. Total deposition of 24-MCF, γ-tocotrienol, and β-sitosterol after application of RBO-NE B3 was 4.14 ± 0.85 nmol/cm^2^, 2.94 ± 0.35 nmol/cm^2^, and 3.75 ± 0.63 nmol/cm^2^, respectively; the corresponding values for the control formulation were 0.72 ± 0.42 nmol/cm^2^, 1.40 ± 0.33 nmol/cm^2^, and 1.30 ± 0.55 nmol/cm^2^, respectively (Fig. 6). The endogenous β-sitosterol level in porcine skin is indicated by the dashed line (0.5 nmol/cm^2^). RBO-NE B3 increased β-sitosterol levels by 7-fold.

**Fig. 7:**
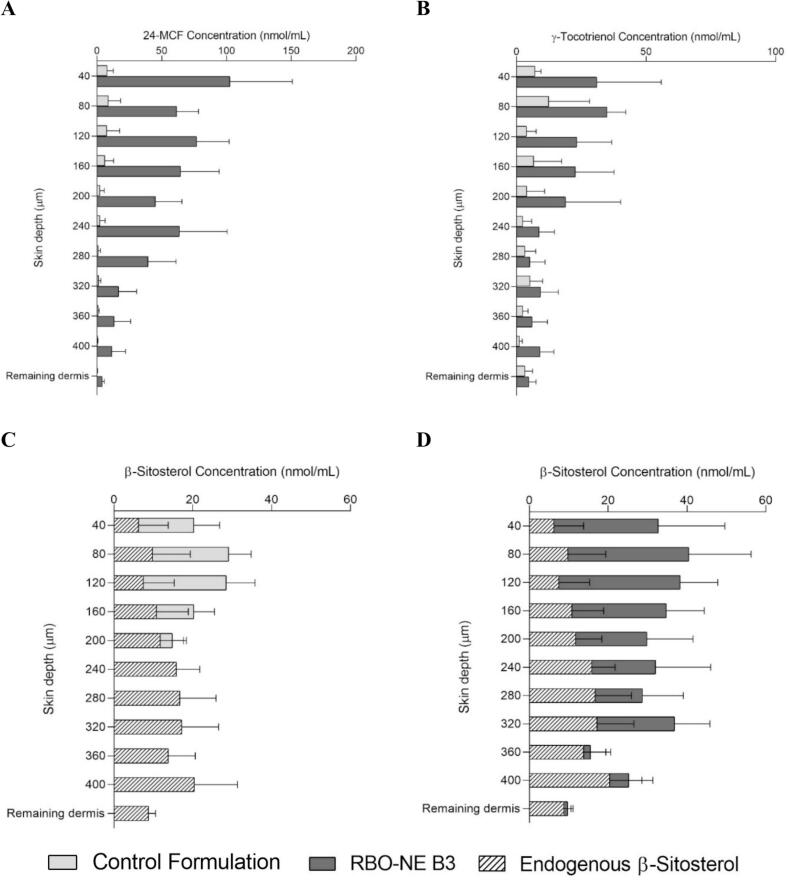
Cutaneous biodistribution profiles in porcine skin: (A) 24-MCF and (B) γ-tocotrienol after application of the control formulation and RBO-NE B3 under infinite dose conditions for 8 h. The corresponding biodistribution profile of β-sitosterol are shown in (C) control formulation and (D) RBO-NE B3, with the endogenous β-sitosterol indicated by hatched lines (Mean ± SD, n = 6).

Biodistribution studies examined how 24-MCF, γ-tocotrienol, and β-sitosterol were distributed at different skin depths (Fig. 7). It was clear that RBO-NE B3 improved the delivery of 24-MCF and γ-tocotrienol to the stratum corneum, epidermis (0–160 μm) and upper dermis (160–240 μm) compared to the control formulation (Fig. 7A and B). For β-sitosterol, it was first necessary to measure the endogenous content in each skin layer. As shown in Fig. 7C and D, endogenous β-sitosterol was present throughout the epidermis and upper dermis (down to 400 mm) – perhaps due to food intake (Fraile et al., 2012). The control formulation was able to deliver β-sitosterol to a depth of 200 mm. In contrast RBO-NE B3 produced a more uniform distribution down to 320 μm.

Previous research has demonstrated that nanoemulsions can enhance the penetration of lipophilic drugs into the skin (El-Leithy et al., 2018; Gokhale et al., 2019; Gul et al., 2022), although the exact mechanism remains unclear and different hypotheses have been proposed: (i) the small globule size, which increases contact area between the drug and skin, (ii) the presence of surfactants and co-surfactants that modify the stratum corneum, destabilizing the lipid bilayer structure, and (iii) the hydrophilic domain of the nanoemulsion, which can hydrate the stratum corneum to enhance drug uptake through the skin (El-Leithy et al., 2018; Nastiti et al., 2017). However, some of these are equally applicable to microemulsisons.

### 3.3 Evaluation of follicular delivery of 24-MCF, γ-tocotrienol and β-sitosterol from RBO-NE B3 in porcine skin under infinite dose conditions

**Fig. 8:**
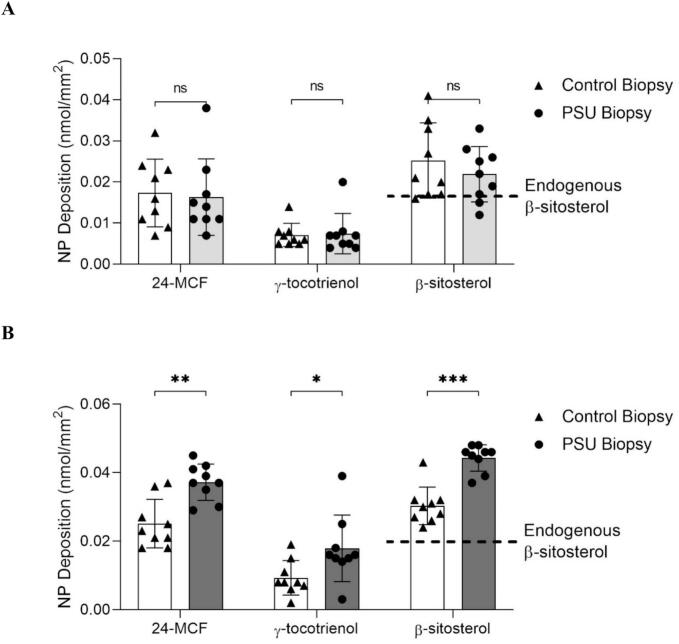
Delivery of 24-MCF, γ-tocotrienol and β-sitosterol to PSU-containing and PSU-free porcine skin biopsies after application of (A) control formulation and (B) RBO-NE B3 under infinite dose conditions for 8 h. The dashed line: endogenous β-sitosterol. (Mean ± SD, *n* = 9, *** *p* < 0.0005, ** *p* < 0.005, * *p* < 0.05, ns = no significant difference, Mann-Whitney test).

Follicular deposition of 24-MCF, γ-tocotrienol, and β-sitosterol after application of RBO-NE B3 and control formulations for 8 h under infinite dose conditions was investigated using PSU-containing biopsies and the results were compared to those obtained with PSU-free biopsy samples in order to estimate the potential for preferential delivery to the PSU (Fig. 8). It was clear that RBO-NE B3 showed higher delivery of 24-MCF, γ-tocotrienol and β-sitosterol to the PSU (0.037 ± 0.005, 0.018 ± 0.01, and 0.044 ± 0.004 nmol/mm^2^, respectively) than in PSU-free biopsies (0.025 ± 0.007, 0.009 ± 0.005, and 0.03 ± 0.006 nmol/mm^2^, respectively). In contrast, there was no difference for the control formulation (*p* > 0.05).

The technique used in this study has been previously applied to investigate the PSU delivery from micelle formulations (Dahmana et al., 2021; Kandekar et al., 2018; Lapteva et al., 2015). The results confirmed that nanoemulsions could enhance the follicular penetration of a highly lipophilic drug. Although the mechanism is not fully understood, flexible nanoemulsion droplets may penetrate via absorption and fusion within the hair follicle infundibulum, as supported by molecular dynamics simulations (Machado et al., 2022).

### 3.4 Evaluation of the cutaneous delivery of 24-MCF, γ-tocotrienol and β-sitosterol from RBO-NE B3 in porcine and human skin under finite dose conditions

**Fig. 9:**
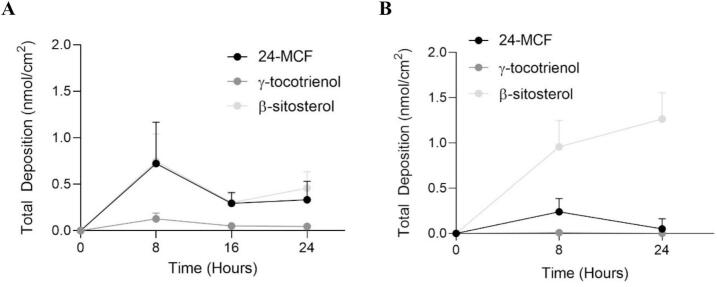
Total deposition of 24-MCF, γ-tocotrienol, and β-sitosterol following a finite dose application of RBO-NE for different time applications on (A) porcine and (B) human skin. The data of β-Sitosterol was shown after subtraction with the endogenous level in both skin (Mean ± SD, *n* = 6).

In vitro experiments conducted under finite dose conditions offer greater relevance and predictive power as they mimic in vivo application more closely. Porcine skin was used in the previous infinite-dose experiment because it is widely considered one of the best surrogates for human skin, providing a general overview of compound distribution due to its similar structure and permeability characteristics (Barbero and Frasch, 2009; Sekkat et al., 2002). Moreover, the possibility to use human skin enhances physiological and anatomical relevance, providing a more realistic model for assessing dermal absorption. In this study, we evaluated the delivery of 24-MCF, γ-tocotrienol and β-sitosterol from RBO-NE B3 in both porcine and human skin under finite dose conditions after formulation application for different durations (Fig. 9). Including both models therefore strengthens the study by enabling comparison between a widely used experimental model and the clinically relevant tissue. The experiments were conducted for 8, 16, and 24 h with porcine skin and for 8 and 24 h with human skin (given its more limited availability) and were performed without occlusion. It was observed that the deposition remained effectively stable after application for 8 h, with no significant difference between results at 8 and 24 h in either porcine or human skin. This can be attributed to the evaporation of water from RBO-NE B3 after application to the skin surface, which results in the formation of a solid residual film on the skin surface. Given the water content, it is envisaged that under finite dose conditions, approximately 90% of the formulation evaporates, thereby limiting the partitioning into the stratum corneum.

After application of RBO-NE B3 for 8 h to porcine skin, the depositions of 24-MCF, γ-tocotrienol, and β-sitosterol were 0.73 ± 0.44 nmol/cm^2^, 0.13 ± 0.06 nmol/cm^2^, and 0.75 ± 0.29 nmol/cm^2^, respectively. For human skin, the corresponding amounts were 0.24 ± 0.15 nmol/cm^2^, 0.01 ± 0.01 nmol/cm^2^ and 0.96 ± 0.29 nmol/cm^2^, respectively. Thus, delivery of 24-MCF appeared to be ∼3-fold lower in human skin. For β-sitosterol, the measured deposition was comparable at 8 h (the result was shown after subtraction of the endogenous level in the respective tissues). However, γ-tocotrienol delivery in human skin was markedly reduced.

**Fig. 10:**
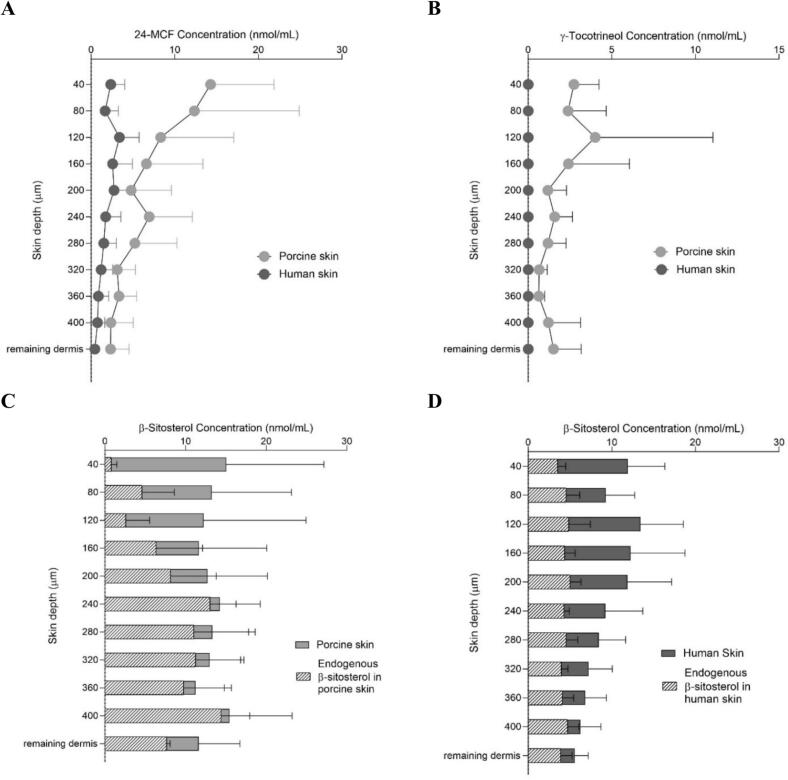
Cutaneous biodistribution profile of (A) 24-MCF, (B) γ-tocotrienol, and (C) β-sitosterol in porcine skin, (D) β-sitosterol in human skin following finite dose application of RBO-NE B3 for 8 h on porcine and human skin.

Further insight was gained from the biodistribution profile of each NP (Fig. 10). The differences in the delivery of 24-MCF and γ-tocotrienol between porcine and human skin were due to superior amounts in the stratum corneum and viable epidermis. Endogenous β-sitosterol was present throughout the epidermis and upper dermis in both porcine and human skin although there were greater amounts in porcine skin. Porcine skin was observed to have twice the endogenous level of β-sitosterol compared to human skin (0.5 nmol/cm^2^ and 0.25 nmol/cm^2^, respectively) and it also showed a different profile compared to human skin. This difference is likely due to the porcine diet (Bhattacharyya et al., 1983; Fraile et al., 2012). These slight differences did not directly impact the deposition of β-sitosterol at 8 h, as the amount of β-sitosterol deposited was comparable in humans and pigs. While porcine skin is the best surrogate for human skin, anatomical and physiological differences – e.g. thinner stratum corneum, different lipid content – can result in slightly higher permeability (Elloso et al., 2025).

Regarding the minimal amount of γ-tocotrienol delivered to both porcine and human skin, it is important to highlight (i) its low concentration in the formulation, which affects interfacial mass transfer (Hadgraft and Lane, 2016), and (ii) as a potent antioxidant it may degrade during or after application (Liu et al., 2021; Rosen et al., 2022). Previous studies have reported that highly active antioxidants are susceptible to oxidation on the skin surface (Thiele et al., 1997). Further studies should consider repeated applications to assess compound accumulation and investigate potential biotransformation by tracking metabolites post-application.

## 4 Conclusions

This study demonstrated that an optimized RBO-based nanoemulsion, RBO-NE B3, was able to enhance the cutaneous delivery of key antioxidant components – 24 MCF, γ-tocotrienol, and β-sitosterol – as compared to a “non-nanoemulsified” control. RBO-NE B3 was also more effective in delivering 24 MCF, γ-tocotrienol, and β-sitosterol to PSU-containing skin samples as compared to control biopsies. Under finite dose conditions, deposition of the three molecules in both porcine and human skin plateaued after 8 h due to formulation evaporation. These findings emphasize the importance of finite dose testing and the impact of formulation transformation as a function of time after application to the skin (Surber and Knie, 2018). Thus, the RBO-based nanoemulsion seemed to be a promising approach for the cutaneous delivery of its antioxidants. More generally, the combination of the cutaneous biodistribution approach with advanced nanoformulation strategies for the delivery of active molecules from complex natural products, could provide a new framework to identify which molecules are responsible for the observed effects by revealing the amounts present in different anatomical layers, in addition to improving the delivery of highly lipophilic natural antioxidants. The findings are expected to guide the development of more effective and targeted dermatological formulations using plant-based bioactives, supporting both therapeutic and cosmeceutical innovations.

## CRediT authorship contribution statement

Erga Syafitri: Investigation, Methodology, Data curation, Validation, Writing – original draft. Aka Yoann-André Kouassi: Data curation, Investigation, Writing – original draft. Claudia Prezioso: Data curation, Validation, Writing – original draft. Yogeshvar N. Kalia: Conceptualization, Resources, Writing – review & editing, Supervision, Funding acquisition.

## Declaration of competing interest

The authors declare that they have no known competing financial interests or personal relationships that could have appeared to influence the work reported in this paper.

## Data Availability

Data will be made available on request.

## Appendix A Supplementary data

Supplementary material: **Cutaneous delivery of bioactive components from a rice bran oil nanoemulsion and their biodistribution in porcine and human skin - Analytical method development and characterization of NP content in RBO** Image 1
