# Supplementary material for: Cutaneous delivery of bioactive components from a rice bran oil nanoemulsion and their biodistribution in porcine and human skin
Source: Int J Pharm X. 2026 Apr 13;11:100543. doi: 10.1016/j.ijpx.2026.100543 (PMC13100242; doi:10.1016/j.ijpx.2026.100543)
Supplement: Supplementary file 1 — Supplementary material: Cutaneous delivery of bioactive components from a rice bran oil nanoemulsion and their biodistribution in porcine and human skin - Analytical method development and characterization of NP content in RBO [file mmc1.docx]

# Supplementary Data

**Cutaneous delivery of bioactive components from a rice bran oil nanoemulsion and their biodistribution in porcine and human skin**

**Erga Syafitri^a,b,c^, Aka Yoann-André Kouassi^a,b^, Claudia Prezioso^d^, Yogeshvar N. Kalia^a,b*^**

^a^ School of Pharmaceutical Sciences, University of Geneva, Geneva, Switzerland

^b^ Institute of Pharmaceutical Sciences of Western Switzerland, University of Geneva, Geneva, Switzerland

^c^ Department of Pharmacy, Faculty of Science, Institut Teknologi Sumatera, South Lampung, Lampung, Indonesia

^d^Department of Food and Drug, Università degli Studi di Parma, Parma, Italy

***Corresponding author**:

Prof. Yogeshvar N. Kalia,

School of Pharmaceutical Sciences, University of Geneva,

CMU - 1 rue Michel-Servet, 1211, Geneva 4, Switzerland.

E-mail: [yogi.kalia@unige.ch](mailto:yogi.kalia@unige.ch)

1. **NP Content in RBO**


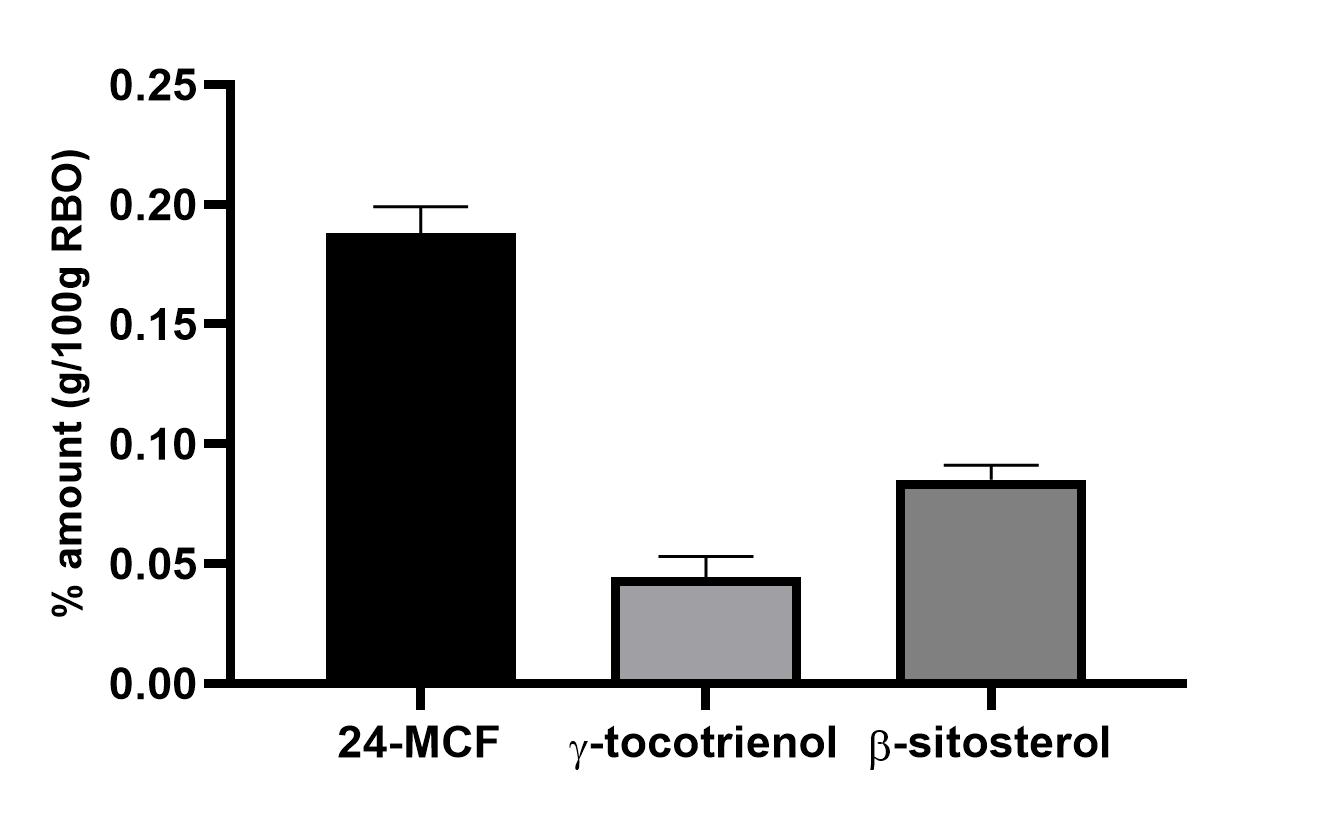


**Figure S1.** NP content in RBO

1. **Development and validation of the UHPLC-MS/MS quantification method**
   1. Development and validation of the quantification method in solvent matrix (ACN)
2. Specificity

**
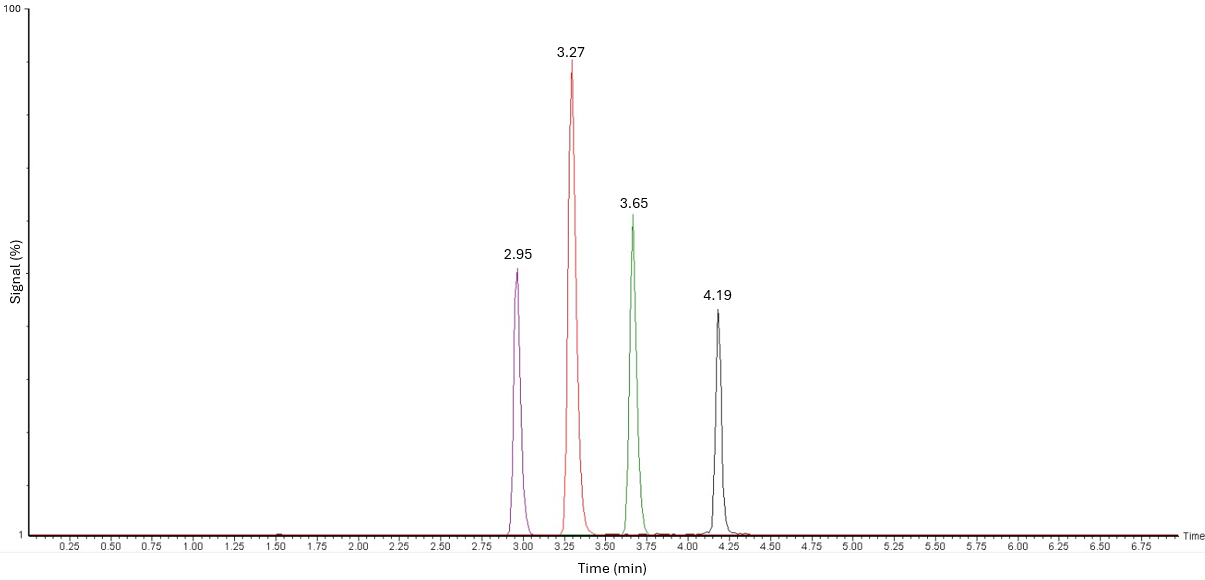
**

**Figure S2.** Chromatogram of γ-tocotrienol (RT = 2.95 min), IS (RT = 3.27 min), β-sitosterol (RT = 3.65 min), and 24-MCF (RT 4.19 min) at a concentration of 50 ng/mL.

1. Linearity, Limit of Detection (LoD) and Limit of Quantification (LoQ)

**Table S1.** Linearity, Limit of Detection (LoD), and Limit of Quantification (LoQ) in a solvent matrix.

|  | 24-MCF | γ-tocotrienol | β-sitosterol |
| --- | --- | --- | --- |
| Range | 1-200 ng/mL | 1-200 ng/mL | 1-200 ng/mL |
| Linearity (r^2^) | 0.9996 | 0.9996 | 0.9991 |
| LoD (ng/mL) | 0.13 | 0.07 | 0.33 |
| LoQ (ng/mL) | 0.5 | 0.1 | 1 |

1. Precision and Accuracy

For this research, precision was quantified in two levels: repeatability (intraday) and intermediate precision (interday).

**Table S2.** Intra and Inter-day accuracy and precision for the UHPLC-MS/MS method in solvent matrix (experimental values are presented as the Mean **±** SD).

1. 24-MCF

| Conc. (ng/mL) | Intra-day | | | Inter-day 1 | | | Inter-day 2 | | |
| --- | --- | --- | --- | --- | --- | --- | --- | --- | --- |
|  | Conc. (mean ± SD) in ng/mL | RSD (%) | Recovery (%) | Conc. (mean ± SD) in ng/mL | RSD (%) | Recovery (%) | Conc. (mean ± SD) in ng/mL | RSD (%) | Recovery (%) |
| 1 | 1.08 ± 0.14 | 11.5 | 107.9 | 0.98 ± 0.02 | 2.4 | 98.3 | 1.01 ± 0.04 | 3.5 | 100.9 |
| 5 | 2.37 ± 0.07 | 2.8 | 118.3 | 2.18 ± 0.18 | 8.3 | 108.8 | 2.15 ± 0.13 | 4.9 | 107.4 |
| 100 | 99.87 ± 5.44 | 5.4 | 99.9 | 103.2 ± 7.6 | 7.4 | 103.2 | 102.5 ± 1.2 | 1.2 | 102.5 |
| 150 | 133.1 ± 0.9 | 0.9 | 88.8 | 141.6 ± 7.8 | 5.5 | 94.4 | 144.2 ± 9.5 | 6.6 | 96.1 |

1. γ-tocotrienol

| Conc. (ng/mL) | Intra-day | | | Inter-day 1 | | | Inter-day 2 | | |
| --- | --- | --- | --- | --- | --- | --- | --- | --- | --- |
|  | Conc. (mean ± SD) in ng/mL | RSD (%) | Recovery (%) | Conc. (mean ± SD) in ng/mL | RSD (%) | Recovery (%) | Conc. (mean ± SD) in ng/mL | RSD (%) | Recovery (%) |
| 1 | 1.14 ± 0.03 | 2.4 | 113.9 | 1.05 ± 0.07 | 7 | 105.1 | 1.07 ± 0.1 | 9.5 | 106.6 |
| 2 | 2.06 ± 0.07 | 3.2 | 102.9 | 2.02 ± 0.03 | 1.3 | 100.8 | 2 ± 0.03 | 1.4 | 99.8 |
| 100 | 98.74 ± 2.1 | 2.1 | 98.7 | 100.7 ± 0.9 | 0.9 | 100.7 | 99.7 ± 2.8 | 2.8 | 99.7 |
| 150 | 133.9 ± 2.7 | 2 | 89.2 | 144.8 ± 7.0 | 4.8 | 96.5 | 142.4 ± 10.1 | 7.1 | 94.7 |

1. β-sitosterol

| Conc. (ng/mL) | Intra-day | | | Inter-day 1 | | | Inter-day 2 | | |
| --- | --- | --- | --- | --- | --- | --- | --- | --- | --- |
|  | Conc. (mean ± SD) in ng/mL | RSD (%) | Recovery (%) | Conc. (mean ± SD) in ng/mL | RSD (%) | Recovery (%) | Conc. (mean ± SD) in ng/mL | RSD (%) | Recovery (%) |
| 1 | 0.97 ± 0.05 | 5.1 | 97 | 0.96 ± 0.05 | 5.7 | 96.2 | 0.95 ± 0.02 | 1.7 | 95 |
| 2 | 2.03 ± 0.08 | 4.1 | 101 | 2.04 ± 0.1 | 4.7 | 101.8 | 2.05 ± 0.09 | 4.3 | 102.3 |
| 100 | 106.9 ± 4.81 | 4.5 | 107 | 103.3 ± 7.4 | 7.2 | 103 | 102.6 ± 5.4 | 5.3 | 102.6 |
| 150 | 151.9 ± 3.5 | 2.3 | 101 | 149.9 ± 1.2 | 0.8 | 100 | 150.2 ± 1.9 | 1.3 | 100.1 |

The RSD criterion is below 15%, and the recovery criterion is between 85-115%, except for LLQC, which is between 80-120%. All the values for 3 NP meet these criteria.

1. Carry Over

The response rate was 15%, indicating no carryover.

- 1. Development and validation of the quantification method in porcine skin matrix

Due to the presence of endogenous β-sitosterol in the porcine skin matrix, all validation was performed using the background-subtraction approach (ICH guideline M10). The blank matrix from the same sources of pig’s ear was used to construct the calibration curve.

1. Specificity

**
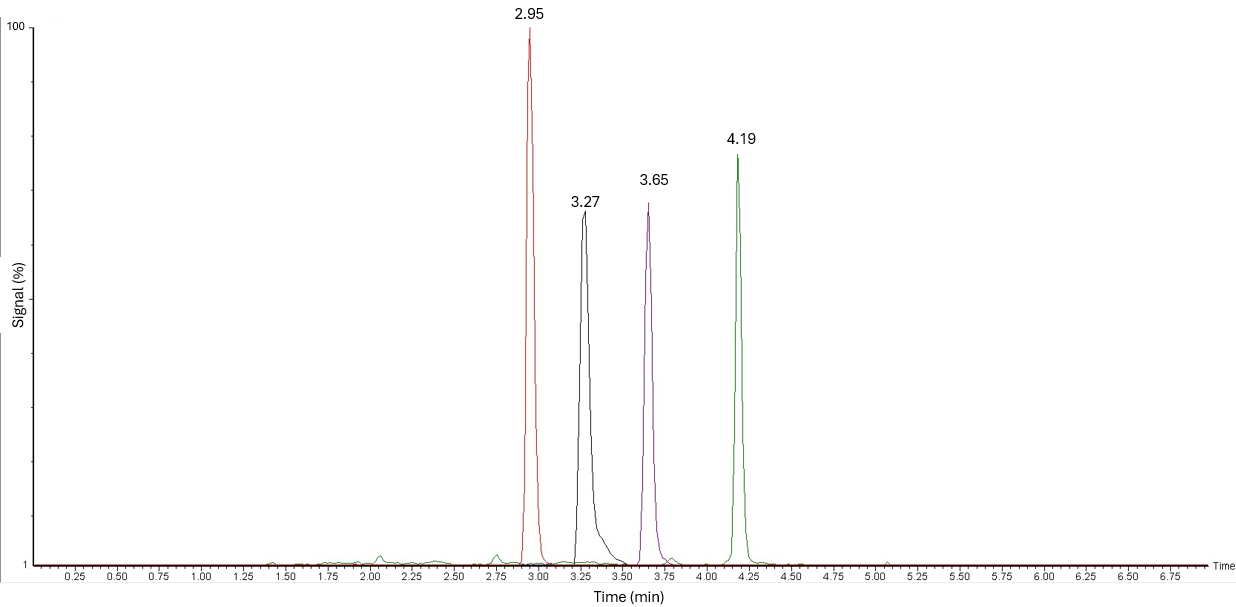
**

**Figure S3.** Chromatogram of γ-tocotrienol (RT = 2.95 min), IS (RT = 3.27 min), β-sitosterol (RT = 3.65 min), and 24-MCF (RT 4.19 min) at a concentration of 100 ng/mL.

1. Linearity, Limit of Detection (LoD) and Limit of Quantification (LoQ)

**Table S3.** Linearity, Limit of Detection (LoD), and Limit of Quantification (LoQ) in porcine skin matrix.

|  | 24-MCF | γ-tocotrienol | β-sitosterol |
| --- | --- | --- | --- |
| Range | 1-200 ng/mL | 1-200 ng/mL | 5-200 ng/mL |
| Linearity (r^2^) | 0.999 | 0.9992 | 0.9991 |
| LoD (ng/mL) | 2.08 | 0.96 | 6.67 |
| LoQ (ng/mL) | 4.15 | 2.29 | 20 |

1. Precision and Accuracy

For this research, precision was quantified in two levels: repeatability (intraday) and intermediate precision (interday).

**Table S4.** Intra and Inter-day accuracy and precision for the UHPLC-MS/MS method in porcine skin matrix (experimental values are presented as the Mean **±** SD).

1. 24-MCF

| Conc. (ng/mL) | Intra-day | | | Inter-day 1 | | | | Inter-day 2 | | |
| --- | --- | --- | --- | --- | --- | --- | --- | --- | --- | --- |
|  | Conc. (mean ± SD) in ng/mL | RSD (%) | Recovery (%) | Conc. (mean ± SD) in ng/mL | RSD (%) | Recovery (%) | Conc. (mean ± SD) in ng/mL | | RSD (%) | Recovery (%) |
| 1 | 1.01 ± 0.10 | 10.3 | 100.6 | 1.00 ± 0.13 | 13.3 | 99.6 | 1.05 ± 0.09 | | 8.7 | 105.5 |
| 10 | 10.46 ± 0.58 | 5.5 | 104.6 | 9.13 ± 1.23 | 13.5 | 91.3 | 8.86 ± 1.33 | | 15 | 88.6 |
| 100 | 94.53 ± 0.41 | 0.4 | 94.5 | 93.6 ± 1.41 | 1.5 | 93.6 | 91.47 ± 3.3 | | 3.6 | 91.5 |
| 200 | 196.5 ± 4.47 | 1.5 | 98.8 | 204.7 ± 21.9 | 7.2 | 101.6 | 201.93 ± 21.6 | | 7.2 | 100.6 |

1. γ-tocotrienol

| Conc. (ng/mL) | Intra-day | | | Inter-day 1 | | | | Inter-day 2 | | |
| --- | --- | --- | --- | --- | --- | --- | --- | --- | --- | --- |
|  | Conc. (mean ± SD) in ng/mL | RSD (%) | Recovery (%) | Conc. (mean ± SD) in ng/mL | RSD (%) | Recovery (%) | Conc. (mean ± SD) in ng/mL | | RSD (%) | Recovery (%) |
| 1 | 0.82 ± 0.05 | 5.6 | 82.3 | 0.94 ± 0.1 | 10.6 | 93.8 | 0.98 ± 0.1 | | 9.7 | 98.3 |
| 10 | 10.14 ± 0.71 | 7 | 101.4 | 10.74 ± 0.3 | 2.8 | 107.4 | 10.68 ± 0.63 | | 5.9 | 106.8 |
| 100 | 98.6 ± 3.8 | 3.9 | 98.6 | 105.5 ± 3.96 | 3.7 | 105.5 | 105.52 ± 9.9 | | 9.5 | 105.5 |
| 200 | 200.7 ± 19.1 | 6.4 | 100.2 | 187.6 ± 14.8 | 5.1 | 95.9 | 196.5 ± 21.3 | | 7.2 | 98.8 |

1. β-sitosterol

| Conc. (ng/mL) | Intra-day | | | Inter-day 1 | | | | Inter-day 2 | | |
| --- | --- | --- | --- | --- | --- | --- | --- | --- | --- | --- |
|  | Conc. (mean ± SD) in ng/mL | RSD (%) | Recovery (%) | Conc. (mean ± SD) in ng/mL | RSD (%) | Recovery (%) | Conc. (mean ± SD) in ng/mL | | RSD (%) | Recovery (%) |
| 20 | 17.5 ± 2.46 | 14 | 87.5 | 14.83 ± 1.79 | 12.1 | 74.1 | 13.44 ± 2.2 | | 16.5 | 67.2 |
| 100 | 103.93 ± 4.3 | 4.1 | 103.9 | 97.73 ± 9.02 | 9.2 | 97.7 | 95.9 ± 7.14 | | 7.4 | 96 |
| 200 | 189.0 ± 12.4 | 4.3 | 96.3 | 210.4 ± 14.7 | 4.7 | 103.5 | 181.3 ± 23.7 | | 8.4 | 93.8 |

The RSD criterion is below 15%, and the recovery criterion is between 85-115%, except for LLQC, which is between 80-120%. All the values for 3 NP meet these criteria.

1. Carry Over

The response rate was 12.5%, indicating no carryover.

- 1. Development and validation of the quantification method in human skin matrix

Due to the presence of endogenous β−sitosterol in porcine skin matrix, all the validation was done using the background subtraction approach (ICH guideline M10). The blank matrix from the same sources of pig’s ear was used to construct the calibration curve.

1. Specificity

**
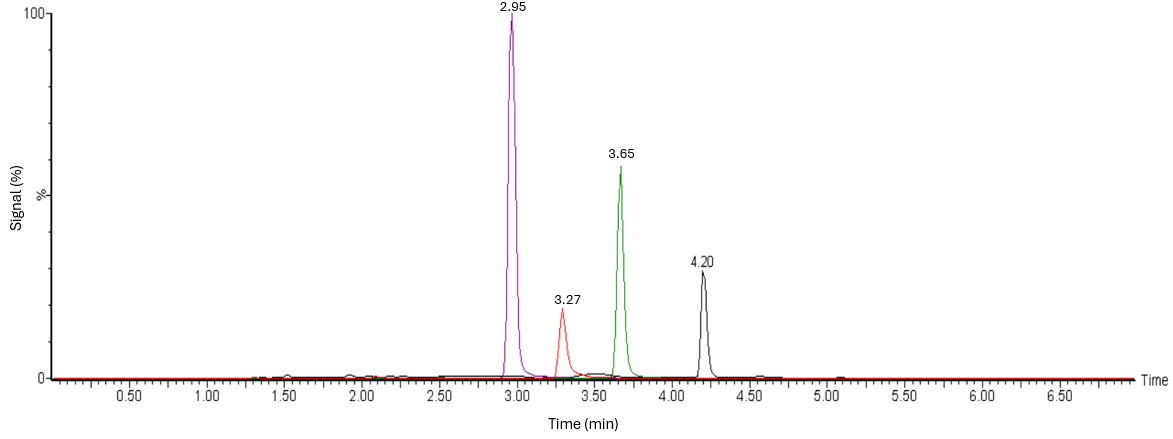
**

**Figure S4.** Chromatogram of γ-tocotrienol (RT = 2.95 min), IS (RT = 3.27 min), β-sitosterol (RT = 3.66 min) and 24-MCF (RT 4.2 min) at a concentration of 100 ng/mL.

1. Linearity, Limit of Detection (LoD) and Limit of Quantification (LoQ)

**Table S5.** Linearity, Limit of Detection (LoD) and Limit of Quantification (LoQ) of 3 NP in human skin matrix.

|  | 24-MCF | γ-tocotrienol | β-sitosterol |
| --- | --- | --- | --- |
| Range | 1-200 ng/mL | 1-200 ng/mL | 5-200 ng/mL |
| Linearity (r^2^) | 0.9996 | 0.9998 | 0.9993 |
| LoD (ng/mL) | 1.85 | 0.07 | 1.67 |
| LoQ (ng/mL) | 5.55 | 0.23 | 5 |

1. Precision and Accuracy

For this research, precision was quantified in two levels: repeatability (intraday) and intermediate precision (interday).

**Table S6.** Intra and Inter-day accuracy and precision for the UHPLC-MS/MS method in human skin matrix (experimental values are presented as the Mean **±** SD).

1. 24-MCF

| Conc. (ng/mL) | Intra-day | | | Inter-day 1 | | | | Inter-day 2 | | |
| --- | --- | --- | --- | --- | --- | --- | --- | --- | --- | --- |
|  | Conc. (mean ± SD) in ng/mL | RSD (%) | Recovery (%) | Conc. (mean ± SD) in ng/mL | RSD (%) | Recovery (%) | Conc. (mean ± SD) in ng/mL | | RSD (%) | Recovery (%) |
| 5 | 5.76 ± 0.47 | 8 | 115.2 | 5.31 ± 0.65 | 12.2 | 106.2 | 5 ± 0.59 | | 11.7 | 100.1 |
| 10 | 10.02 ± 0.36 | 4 | 100.2 | 10.39 ± 0.5 | 4.8 | 103.9 | 10.19 ± 0.15 | | 1.4 | 101.9 |
| 100 | 105.21 ± 5.7 | 5 | 105.2 | 109.26 ± 3.32 | 3 | 109.3 | 100.41 ± 1.86 | | 1.9 | 100.4 |
| 150 | 142.2 ± 11.7 | 8 | 94.8 | 150.22 ± 1.22 | 0.8 | 100.1 | 150.94 ± 4.12 | | 2.7 | 100.6 |

1. γ-tocotrienol

| Conc. (ng/mL) | Intra-day | | | Inter-day 1 | | | | Inter-day 2 | | |
| --- | --- | --- | --- | --- | --- | --- | --- | --- | --- | --- |
|  | Conc. (mean ± SD) in ng/mL | RSD (%) | Recovery (%) | Conc. (mean ± SD) in ng/mL | RSD (%) | Recovery (%) | Conc. (mean ± SD) in ng/mL | | RSD (%) | Recovery (%) |
| 2 | 1.87 ± 0.03 | 1.8 | 93 | 1.9 ± 0.06 | 3 | 95.2 | 1.88 ± 0.05 | | 2.6 | 94.2 |
| 5 | 5.22 ± 0.12 | 2.2 | 104 | 4.93 ± 0.15 | 3 | 98.5 | 5.21 ± 0.11 | | 2.1 | 104.1 |
| 100 | 104.26 ± 2.2 | 2.1 | 104 | 106.33 ± 7.64 | 7.2 | 106.3 | 105.65 ± 3.56 | | 3.4 | 105.7 |
| 150 | 150.98 ± 2.7 | 1.8 | 101 | 158.94 ± 10.4 | 6.5 | 106 | 154.47 ± 6.47 | | 4.2 | 103 |

1. β-sitosterol

| Conc. (ng/mL) | Intra-day | | | Inter-day 1 | | | | Inter-day 2 | | |
| --- | --- | --- | --- | --- | --- | --- | --- | --- | --- | --- |
|  | Conc. (mean ± SD) in ng/mL | RSD (%) | Recovery (%) | Conc. (mean ± SD) in ng/mL | RSD (%) | Recovery (%) | Conc. (mean ± SD) in ng/mL | | RSD (%) | Recovery (%) |
| 5 | 5.77 ± 0.25 | 4.3 | 115 | 5.3 ± 0.45 | 8.4 | 106.1 | 5.39 ± 0.41 | | 7.6 | 107.8 |
| 10 | 10.77 ± 0.36 | 3.4 | 108 | 10.22 ± 0.43 | 4.2 | 102.2 | 10.49 ± 0.49 | | 4.6 | 104.9 |
| 100 | 98.61 ± 1.19 | 1.2 | 99 | 99.87 ± 2.16 | 2.2 | 99.9 | 101.87 ± 3.01 | | 3 | 101.9 |

The RSD criterion is below 15%, and the recovery criterion is between 85-115%, except for LLQC, which is between 80-120%. All the values for 3 NP meet these criteria.

1. Carry Over

The response rate was 13.6%, indicating no carryover.

1. **Validation of the NP extraction procedure from RBO**

RBO 20 mg was spiked with different concentrations of stock solution, resulting in expected values (2 ng/mL, 40 ng/mL, and 100 ng/mL). The recovery was calculated for each concentration.

**Table S7.** Extraction recovery of 3 NP from RBO.

|  | Recovery (% ± SD) | | |
| --- | --- | --- | --- |
|  | Low Concentration (2 ng/mL) | Middle Concentration  (40 ng/mL) | High Concentration  (100 ng/mL) |
| 24-MCF | 119.6 ± 21.7 | 111.7 ± 14.1 | 108.5 ± 6.7 |
| γ-tocotrienol | 97 ± 16.6 | 124.3 ± 12 | 104.6 ± 1.7 |
| β-sitosterol | 105.0 ± 19.3 | 91.9 ± 3.7 | 89.0 ± 2.5 |

1. **Endogenous β-sitosterol level in porcine and human skin**

**Table S8.** Endogenous β-sitosterol level in porcine skin

| Pig Donor | Sample | Concentration in the skin (ng/mg skin) | Amount in the skin per area (ng/cm^2^) | Amount in the skin per area (nmol/cm^2^) |
| --- | --- | --- | --- | --- |
| P1 | P1_a | 2.79 | 207.26 | 0.5 |
|  | P1_b | 2.57 | 191.9 | 0.46 |
|  | P1_c | 2.32 | 201.16 | 0.49 |
| P2 | P2_a | 2.54 | 180.7 | 0.44 |
|  | P2_b | 2.86 | 211.42 | 0.51 |
|  | P2_c | 1.9 | 204.58 | 0.49 |
| P3 | P3_a | 2.67 | 197.97 | 0.48 |
|  | P3_b | 2.75 | 205.32 | 0.50 |
|  | P3_c | 2.33 | 201.86 | 0.49 |
| P4 | P4_a | 2.35 | 155.35 | 0.37 |
|  | P4_b | 2.20 | 224.26 | 0.54 |
|  | P4_c | 2.15 | 148.91 | 0.36 |
| P5 | P5_a | 2.94 | 218.25 | 0.53 |
|  | P5_b | 3.03 | 225.74 | 0.54 |
|  | P5_c | 2.22 | 191.89 | 0.46 |

Average of endogenous β-sitosterol level in porcine skin = 0.48 ± 0.05 nmol/cm^2^

**Table S9.** Endogenous β-sitosterol level in human skin

| Human Donor | Samples | Concentration in the sample (ng/mg) | Amount in the skin per area (ng/cm^2^) | Amount in the skin per area (nmol/cm^2^) |
| --- | --- | --- | --- | --- |
| HS-165-4 (20/03/2025) | HS-165-a | 1.12 | 99.12 | 0.24 |
|  | HS-165-b | 1.14 | 87.84 | 0.21 |
|  | HS-165-c | 0.89 | 134.88 | 0.33 |
| HS-166-7 (27/03/2025) | HS-166-a | 1.02 | 133.26 | 0.32 |
|  | HS-166-b | 1.11 | 115.37 | 0.28 |
|  | HS-166-c | 0.75 | 104.97 | 0.25 |
| HS-167-29 (14/04/2025) | HS-167-a | 0.68 | 66.64 | 0.16 |
|  | HS-167-b | 0.78 | 84.64 | 0.20 |
|  | HS-167-c | 0.72 | 88.53 | 0.21 |

Average of endogenous β-sitosterol level in human skin = 0.25 ± 0.05 nmol/cm^2^

1. **Validation of washing method**

**Table S10.** Validation of the washing method

| NP | Concentration experimental (ng/mL) |
| --- | --- |
| 24-MCF | 4.13 ± 1.69 |
| γ-tocotrienol | 0.22 ± 0.02 |
| β-sitosterol | - 1. ± 0.02 |

The concentration detected below LoQ

1. **Validation of NP extraction procedure from porcine and human skin.**
   1. Validation of NP extraction procedure for the total deposition sample

**Table S11.** Extraction recovery of NP from porcine skin.

|  | Recovery (% ± SD, n=3) | | |
| --- | --- | --- | --- |
|  | Low Concentration (10 ng/mL) | Middle Concentration (100 ng/mL) | High Concentration (200 ng/mL |
| 24-MCF | 110 ± 7.6 | 111.7 ± 16.7 | 105.8 ± 4.7 |
| γ-tocotrienol | 95 ± 9.5 | 87.5 ± 23.3 | 98.0 ± 5.4 |
| β-sitosterol | 108.0 ± 5.3 | 113.7 ± 6.7 | 112.5 ± 0.9 |

**Table S12.** Extraction recovery of 3 NP from human skin.

|  | Recovery (% ± SD, n=3) | | |
| --- | --- | --- | --- |
|  | Low Concentration (10 ng/mL) | Middle Concentration (100 ng/mL) | High Concentration (200 ng/mL |
| 24-MCF | 112.7 ± 8.3 | 90.3 ± 8.4 | 96.3 ± 11.2 |
| γ-tocotrienol | 110.3 ± 23.5 | 80.7 ± 3.5 | 86.7 ± 14.4 |
| β-sitosterol | 114 ± 13 | 94 ± 9.6 | - 1. ± 10.9 |

- 1. Validation of NP extraction procedure for biodistribution samples

**Table S13.** Extraction recovery of NP from porcine and human skin from biodistribution samples.

|  | Recovery (% ± SD) of low concentration (10 ng/mL), n= 3 | |
| --- | --- | --- |
|  | Porcine skin | Human skin |
| 24-MCF | 10.7 ± 5.3 | 100.7 ± 10.0 |
| γ-tocotrienol | 87.3 ± 13.5 | 88.7 ± 6.4 |
| β-sitosterol | 97 ± 5.4 | - 1. ± 6.4 |

1. **Screening of the surfactant and co-surfactant mixtures**

| (A) | (B) | (C) |
| --- | --- | --- |
| 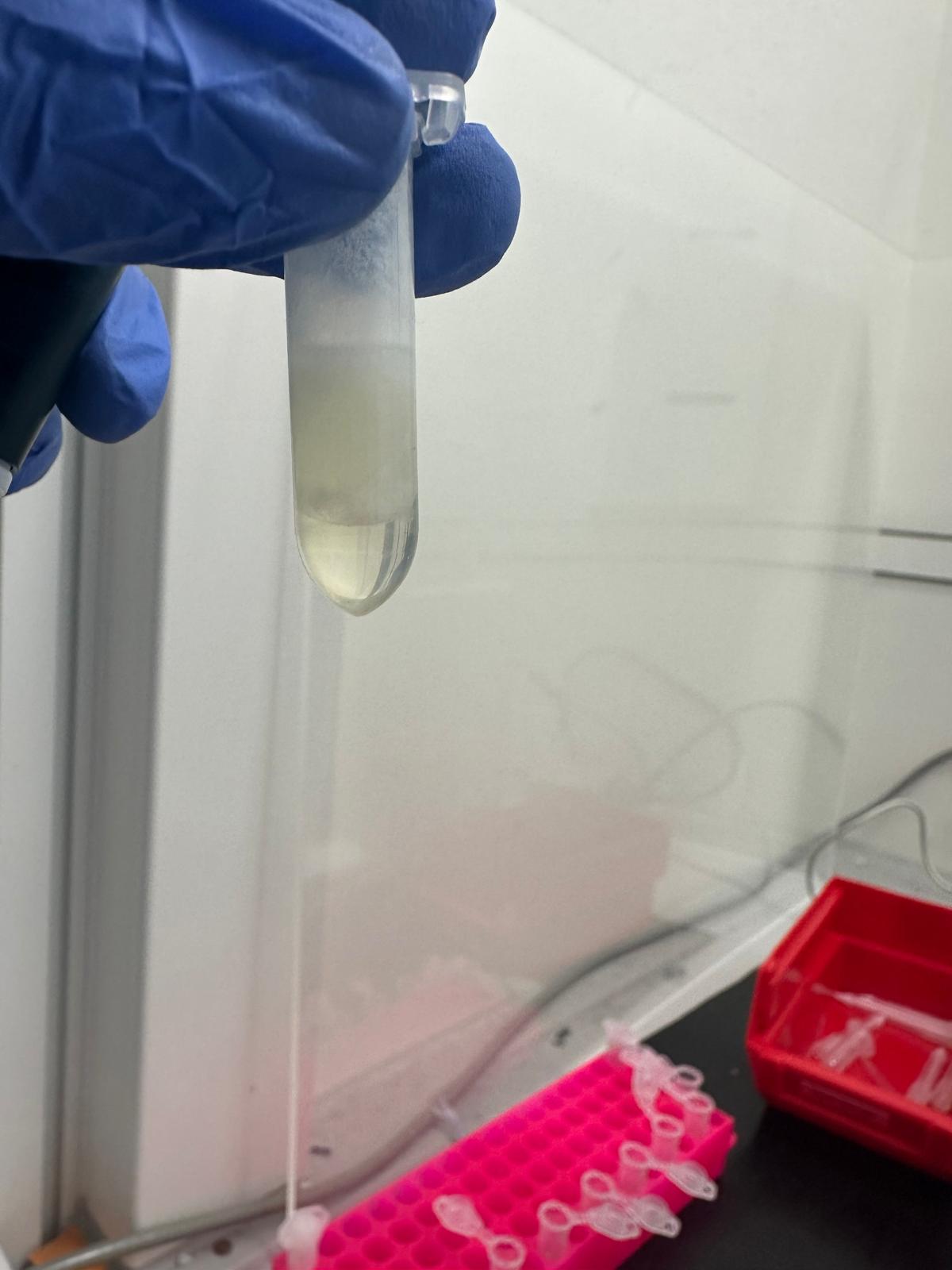 | 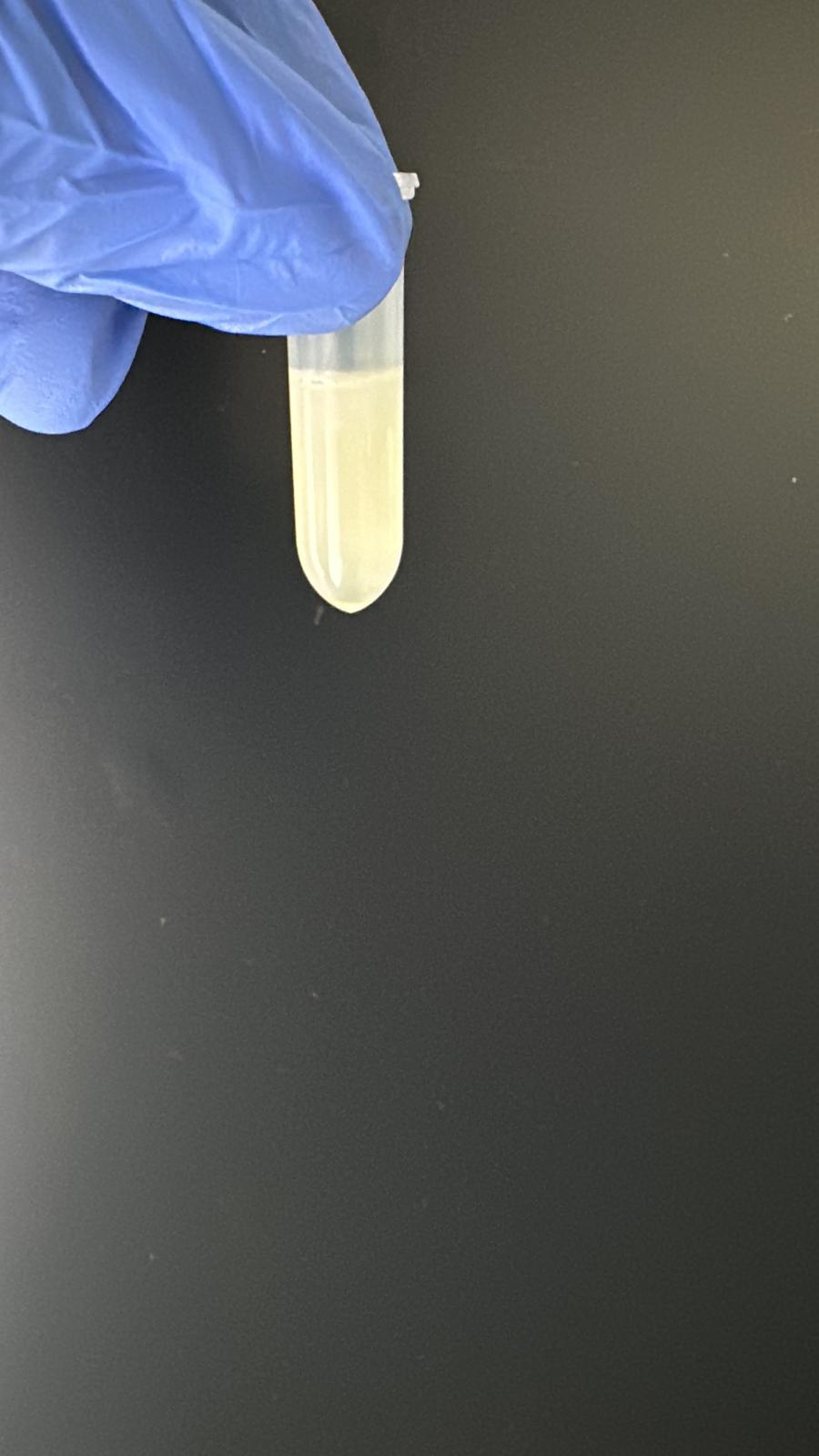 | 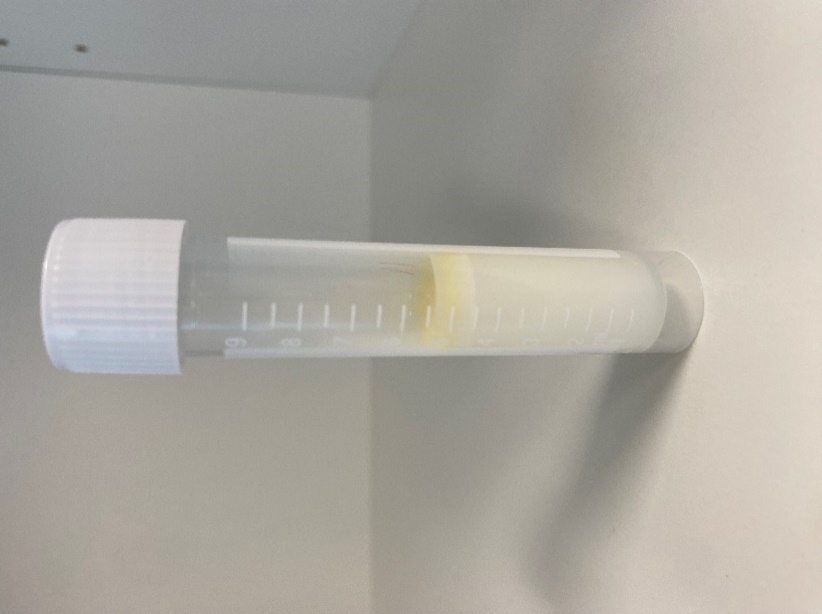 |

**Figure S5.** Physical observation of RBO emulsion with different surfactant and co-surfactant mixture (4:1 ratio) using the water titration method. (A) Labrasol : Transcutol P; (B) Cremophor EL: sorbitan oleate, and (C) Tween 80 : sorbitan oleate.

1. **Determination of pH of RBO-NE B3 over 3 months**

**Table S14.** pH value of RBO-NE B3 over 3 months observation.

| Time (months) | pH |
| --- | --- |
| 0 | 4.4 |
| 1 | 4.5 |
| 2 | 4.8 |
| 3 | 4.7 |
